# Complement C3aR depletion reverses HIF-1**α**–induced metabolic impairment and enhances microglial response to A**β** pathology

**DOI:** 10.1172/JCI167501

**Published:** 2023-06-15

**Authors:** Manasee Gedam, Michele M. Comerota, Nicholas E. Propson, Tao Chen, Feng Jin, Meng C. Wang, Hui Zheng

**Affiliations:** 1 Huffington Center on Aging,; 2 Translational Biology and Molecular Medicine Graduate Program,; 3 Department of Molecular and Human Genetics,; 4 Howard Hughes Medical Institute, Baylor College of Medicine, Houston Texas, USA.

**Keywords:** Neuroscience, Alzheimer disease, Complement, Mouse models

## Abstract

Microglia are the major cell type expressing complement C3a receptor (C3aR) in the brain. Using a knockin mouse line in which a Td-tomato reporter is incorporated into the endogenous *C3ar1* locus, we identified 2 major subpopulations of microglia with differential C3aR expression. Expressing the Td-tomato reporter on the *APP*^NL-G-F^–knockin (APP-KI) background revealed a significant shift of microglia to a high-C3aR-expressing subpopulation and they were enriched around amyloid β (Aβ) plaques. Transcriptomic analysis of C3aR-positive microglia documented dysfunctional metabolic signatures, including upregulation of hypoxia-inducible factor 1 (HIF-1) signaling and abnormal lipid metabolism in APP-KI mice compared with wild-type controls. Using primary microglial cultures, we found that *C3ar1*-null microglia had lower HIF-1α expression and were resistant to hypoxia mimetic–induced metabolic changes and lipid droplet accumulation. These were associated with improved receptor recycling and Aβ phagocytosis. Crossing *C3ar1*-knockout mice with the APP-KI mice showed that C3aR ablation rescued the dysregulated lipid profiles and improved microglial phagocytic and clustering abilities. These were associated with ameliorated Aβ pathology and restored synaptic and cognitive function. Our studies identify a heightened C3aR/HIF-1α signaling axis that influences microglial metabolic and lipid homeostasis in Alzheimer disease, suggesting that targeting this pathway may offer therapeutic benefit.

## Graphical abstract

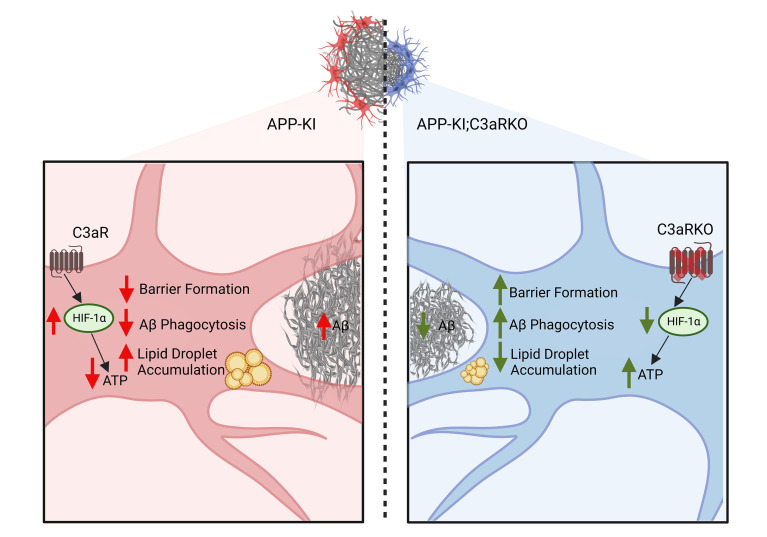

## Introduction

Alzheimer disease (AD), the leading cause of dementia in the elderly, is pathologically characterized by the accumulation of amyloid β (Aβ) plaques and intracellular tau tangles and accompanying neuroinflammation associated with reactive microglia and astrocytes (1, 2). In addition, increasing evidence implicates metabolic deficits as a contributor to AD pathogenesis (3–7).

The mammalian brain has a high energy demand, emphasizing the need for efficient ATP production. Microglia, the major immune cells and the primary phagocytes in the brain, consume ATP to efficiently execute their functions such as phagocytosis, immune surveillance, synaptic maintenance, and trophic support. The metabolically adaptive state of microglia allows them to primarily depend on oxidative phosphorylation (OXPHOS) for their ATP production (8, 9), but also switch to glycolysis in response to high energy needs (10). Hypoxia-inducible factor 1α (HIF-1α), a subunit of HIF-1, is an essential molecule in oxygen sensing and a canonical modulator of metabolic reprogramming. HIF-1 acts on mRNA synthesis to induce the production of glycolytic enzymes and glucose transporters such as pyruvate dehydrogenase kinase 1 (PDK1) and phosphofructokinases (PFK2 and PFKL) to increase the rate of glycolysis and inhibit aerobic respiration. Acute activation of HIF-1α signaling results in the rapid metabolic switch from OXPHOS to anaerobic glycolysis (11–14), allowing fast production of ATP in response to high energy demand. However, in contrast to the 36 ATP per glucose produced by OXPHOS, this process produces only 2 ATP per glucose molecule, which is not sustainable in chronic conditions. Thus, sustained HIF-1 activation leads to compromised cellular bioenergetics and metabolic function.

Microglia display dynamic changes in response to Aβ pathology. Activated microglia cluster around Aβ plaques to form a protective barrier and are also responsible for Aβ uptake and clearance (15). However, microglia under chronic Aβ exposure become reactive and neuroinflammatory (5), exhibiting altered HIF-1 and other metabolic pathways (3, 5, 16, 17) and their response to Aβ pathology.

A major driver of neuroinflammation is the complement pathway, an evolutionarily conserved innate immune signaling mechanism (18, 19). Genetic and functional studies have linked multiple components of the complement pathway to late-onset AD (20–24). C3, a central component of complement signaling, is cleaved by C3 convertase to form C3a, which interacts with its receptor C3aR, predominantly found on tissue macrophages and microglia in the brain, to elicit downstream responses (18). Generally regarded as a mediator of inflammation, recent evidence also implicates C3/C3aR signaling in regulating cellular metabolic processes (25–28). We and others have shown that levels of C3 and C3aR in the brain are elevated in aging and AD mouse models as well as in patients with AD and that their inactivation conferred protection against age-associated functional decline and AD neuropathology (22, 29–32). However, the downstream mechanisms underlying C3aR’s effects are not well understood.

By crossing a C3aR reporter line in which Td-tomato was integrated into the endogenous *C3ar1* locus (33) with *APP*^NL-G-F^–knockin (APP-KI) mice, which carries a humanized Aβ sequence with Swedish, Iberian, and Arctic mutations (34), we found that C3aR was highly enriched in microglia surrounding Aβ plaques. These microglia displayed a compromised metabolic state, with elevated HIF-1α signaling and altered lipid metabolic profiles. Mechanistically, we provide data showing that HIF-1α acted downstream of C3aR to influence microglial bioenergetics and metabolic fitness. Microglia lacking C3aR are resistant to hypoxia mimetic–induced functional impairments, including reduced phagocytic receptor recycling and lipid droplet accumulation. Ablation of C3aR in APP-KI mice restored the lipid signatures and improved the microglial barrier and phagocytic function in response to Aβ pathology, resulting in reduced plaque formation and improved cognition.

## Results

### C3aR-positive microglia display upregulated HIF-1 and altered lipid metabolic profiles.

**Figure 1:**
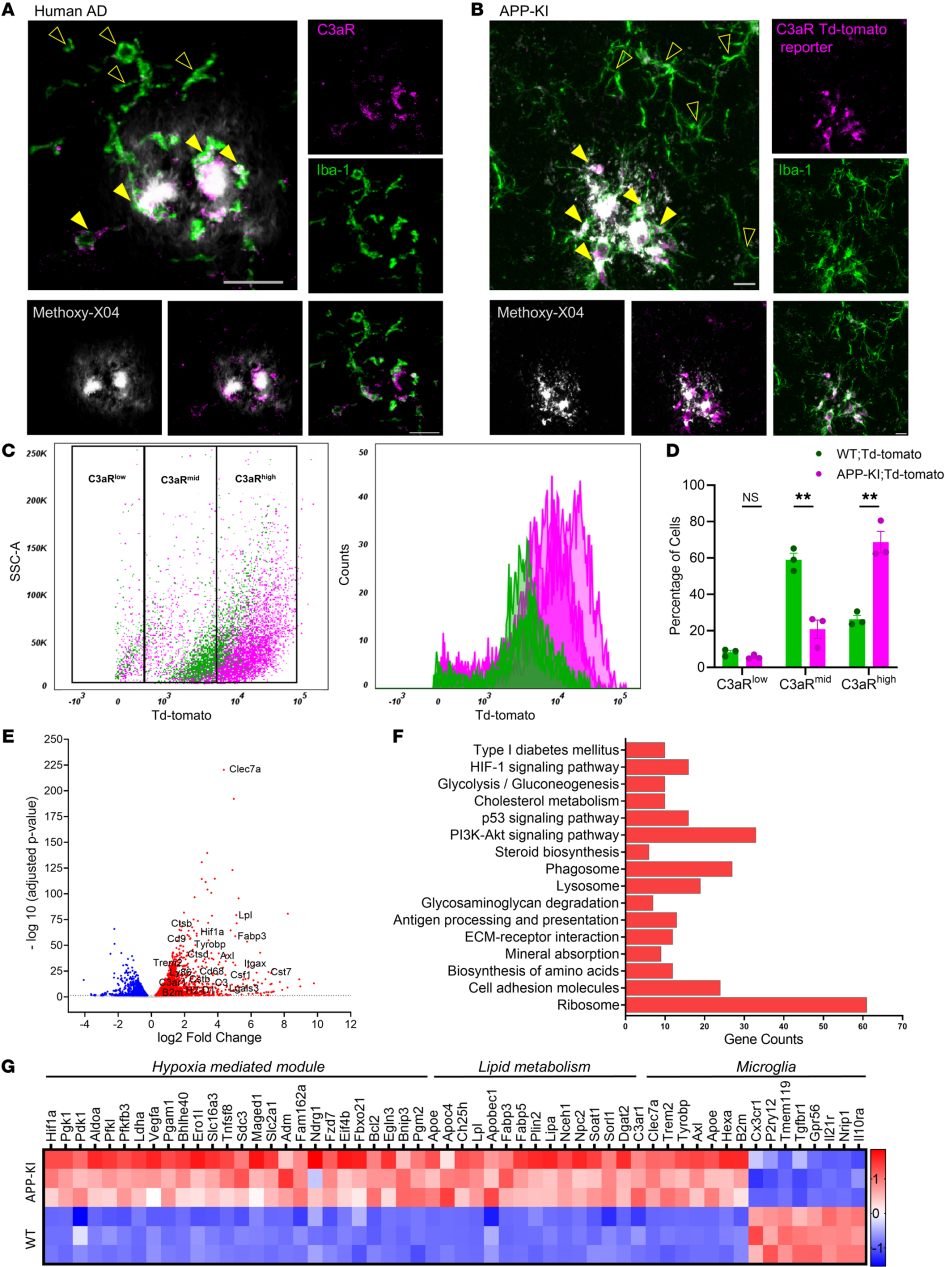
Plaque-associated microglia express C3aR and display a compromised metabolic profile. (**A**) Representative images of human tissues from AD patients stained for C3aR (magenta), Iba-1 (green), and plaques (gray) showing higher C3aR expression in microglia proximal to the plaque (solid yellow arrowheads) compared with microglia distal from the plaque (hollow yellow arrowheads). Scale bar: 10 μm. (**B**) Representative images from 9-month-old APP-KI mice crossed with C3aR Td-tomato reporter (magenta) stained for Iba-1 (green) and plaques (gray). Solid arrowheads mark higher-C3aR-expressing microglia proximal to the plaque compared with microglia distal from the plaque (hollow yellow arrowheads) Scale bar: 10 μm. (**C**) Left: Flow cytometry plot displaying the distribution of microglia from 9-month-old WT and APP-KI mice based on C3aR expression. Right: Histogram depicting the distribution of microglia based on C3aR expression. (**D**) Bar graph showing the percentage of C3aR expression in WT and APP-KI mice. Data presented as mean ± SEM. NS, nonsignificant. **P < 0.01 by 2-sided Student’s *t* test. (**E**) Volcano plot from RNA-seq of C3aR^+^ cells showing upregulated genes (red) and downregulated genes (blue) in 9-month-old APP-KI compared with WT mice. (**F**) Pathway analysis using KEGG revealing significantly altered metabolic and HIF-1 signaling pathways in APP-KI mice compared with WT (*P*-value cutoff: 0.05). (**G**) Heatmap showing altered lipid and hypoxia modules in C3aR^+^ microglia from APP-KI mice compared with WT.

Microglia undergo dynamic changes in response to Aβ pathology, exhibiting molecular, morphological, and functional changes and subtype heterogeneity. Complement C3aR is known to be preferentially expressed in microglia in the brain, where its levels are elevated in AD conditions (29). To gain further understanding of the relationship between microglial C3aR and Aβ, we costained postmortem human AD brain tissues with anti-C3aR and anti–Iba-1 antibodies and found that Iba-1–positive microglia proximal to methoxy-X04–positive plaques displayed much higher C3aR expression compared with microglia distal from the plaques (Figure 1A). To visualize C3aR in mouse brains, we crossed the C3aR-Td-tomato reporter with the APP-KI mice, which develop Aβ plaque pathology at around 3 to 4 months, with synapse loss and cognitive deficits detectable at 6 months (34). Analysis of the mice at 9 months of age showed that, like human AD samples, microglia surrounding the Aβ plaques displayed higher C3aR staining compared with those not associated with the plaques (Figure 1B). To explore the heterogeneity further, we sorted the CD45^+^ and CD11b^+^ microglia from 9-month-old wild-type (WT) and APP-KI mice crossed with the Td-tomato C3aR reporter line and profiled C3aR expression based on Td-tomato intensity (Supplemental Figure 1, A–C; supplemental material available online with this article; https://doi.org/10.1172/JCI167501DS1) (33). In WT mice, we detected a small percentage of cells with either low C3aR (C3aR^lo^) or high (C3aR^hi^) expression, while the majority of microglia exhibited modest levels of C3aR (C3aR^mid^) (Figure 1, C and D). Consistent with higher C3aR expression in response to Aβ pathology, there was a dramatic shift of the C3aR^mid^ population to C3aR^hi^ in APP-KI mice. Interestingly, the C3aR^lo^ population remained unchanged. Similar results were also observed in microglia of 5xFAD mice (Supplemental Figure 1, D–F).

To understand the molecular changes downstream of C3aR, we performed RNA-sequencing (RNA-seq) of Td-tomato–sorted, C3aR-expressing microglia from the WT and APP-KI mice (Supplemental Table 1). Principal component analysis showed distinct clustering of the 2 sample groups (Supplemental Figure 2A). A total of 2445 genes were upregulated, and 1414 genes were downregulated in APP-KI compared with WT. As expected, we found upregulation of reactive microglial genes and downregulation of homeostatic genes that have been previously reported in plaque-associated microglia (35–37) (Figure 1E and Supplemental Figure 2B). Intriguingly, KEGG pathway analysis of dysregulated genes revealed an altered metabolic profile in APP-KI microglia compared with WT, with the HIF-1α signaling pathway as one of the most significantly changed pathways (*P* = 0.0144; Figure 1F). A similar relationship between C3aR and HIF-1α in microglial cells was also found by analyzing a published single-cell transcriptomic data set (GEO GSE127893) of WT and APP-KI mice (Supplemental Figure 2, C and D) (37). Besides the HIF-1α pathway, genes associated with lipid metabolic pathways were also found to be upregulated in APP-KI microglia compared with WT controls (Figure 1G and Supplemental Table 2). Together, these results demonstrate that C3aR levels were elevated in microglia surrounding Aβ plaques and these microglia exhibited altered metabolic profiles exemplified by the upregulation of HIF-1α and lipid signaling pathways.

### Absence of C3aR dampens HIF-1α–induced metabolic and functional perturbations.

**Figure 2:**
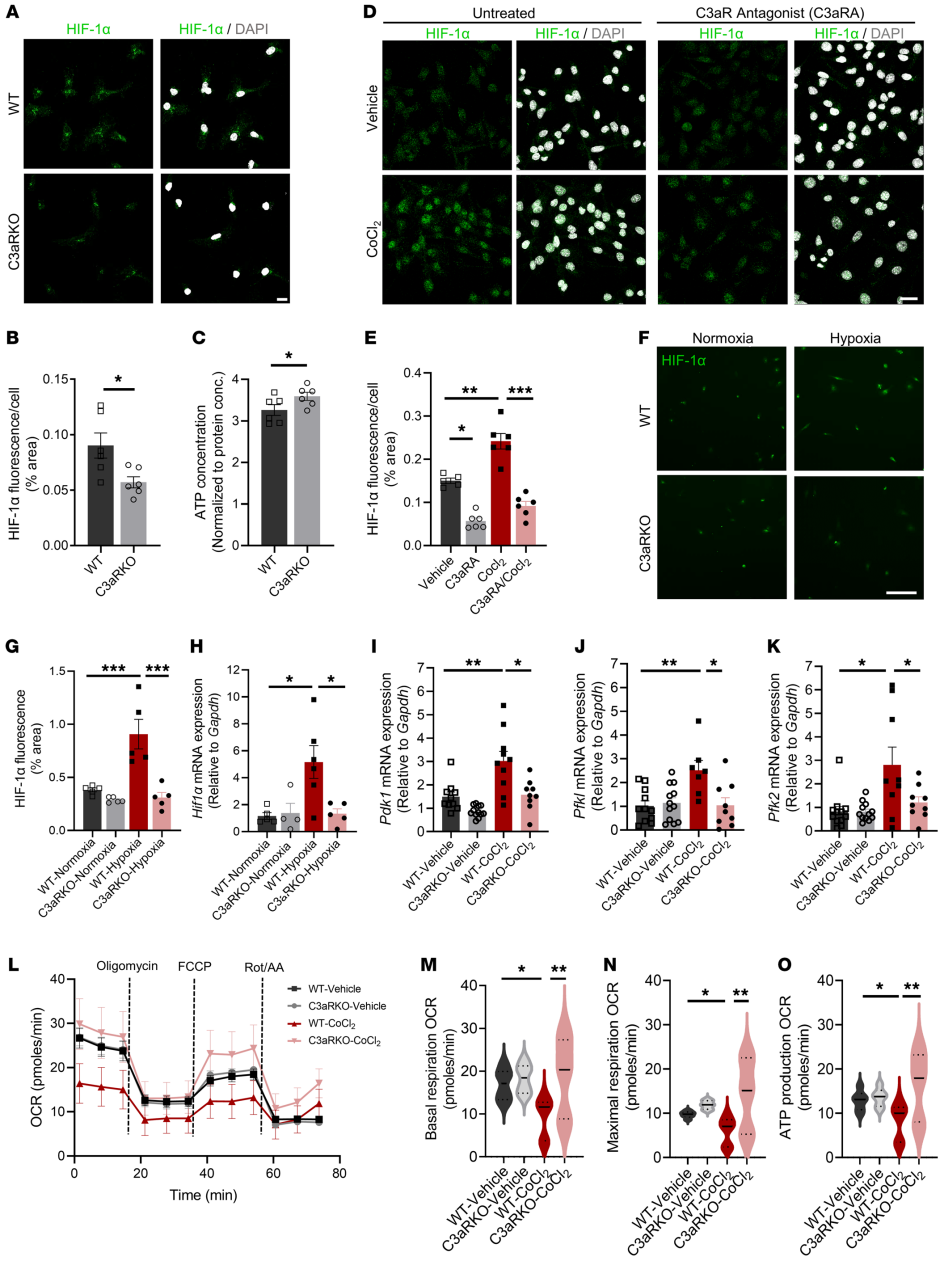
Absence of C3aR dampens HIF-1α–induced metabolic changes. (**A**) Representative images of HIF-1α immunofluorescence (green) and DAPI (gray) in WT and C3aR-KO primary microglial cultures. Scale bar: 15 μm. (**B**) Quantification of percentage area of HIF-1α fluorescence per cell (*n* = 180–198 cells from 6 replicates/group). (**C**) ATP concentration using luminescence assay in WT and C3aR-KO cells (*n* = 6 replicates/group). (**D**) Representative images of HIF-1α (green) and DAPI (gray) in BV-2 cells pretreated with 10 μM C3aR antagonist (C3aRA) prior to treatment with 100 μM CoCl_2_. Scale bar: 100 μm. (**E**) Quantification of HIF-1α expression per cell in BV-2 cells treated with vehicle, 10 μM C3aRA, 100 μM CoCl_2_, or C3aRA + CoCl_2_ (*n* = 250–270 cells from 6 replicates/group). (**F**) Representative images showing HIF-1α (green) immunofluorescence in WT and C3aR-KO primary microglia cells placed in hypoxia chamber (1% O_2_) for 24 hours compared with primary microglial cells placed in normoxia (21% O_2_). Scale bar: 15 μm. (**G**) Quantification of percentage area of HIF-1α fluorescence in WT and C3aR-KO cells exposed to hypoxic or normoxic conditions. (**H**) qPCR analysis of *Hif1a* in WT and C3aR-KO cells exposed to hypoxic or normoxic conditions. (**I**–**K**) qPCR analysis of HIF-1α targets *Pdk1* (**I**), *Pfkl* (**J**), and *Pfk2* (**K**) in response to 100 μM CoCl_2_ treatment in primary microglial cells. (**L**) Oxygen consumption rate (OCR) plot from Seahorse Mito Stress assay performed on WT and C3aR-KO cells with and without 24-hour CoCl_2_ treatment. Violin plots showing the quantification of basal OCR (**M**), maximal OCR (**N**), and ATP production OCR (**O**) across the 4 treatment groups. For all panels, data are presented as mean ± SEM (**B**–**E** and **G**–**L**) or median and quartile range (**M**–**O**). *P < 0.05; **P < 0.01; ***P < 0.001 by 2-sided Student’s *t* test (**B** and **C**) or 1-way ANOVA with Tukey’s correction (**E**, **G**–**K**, and **M**–**O**). Each experiment was repeated 2–3 times.

Chronic exposure to hypoxia has been shown to compromise microglial metabolism and energy production (16). Our studies described above established a positive correlation between C3aR and HIF-1α signaling pathways. To assess a functional role of C3aR in HIF-1α expression and regulation, we prepared primary microglial cultures from littermate WT and *C3ar1*-knockout (C3aR-KO) mice (23). Immunostaining using an anti–HIF-1α antibody showed reduced HIF-1α staining in C3aR-KO cells (Figure 2, A and B, and Supplemental Figure 2E). This was associated with higher ATP concentrations (Figure 2C). Reduced HIF-1α expression was also detected by treating BV-2 microglia-like cells with a C3aR antagonist (C3aRA) (Figure 2, D and E; compare vehicle vs. C3aRA), supporting a direct regulation of HIF-1α by C3aR. Treating the BV-2 cells with cobalt chloride (CoCl_2_), a hypoxia mimetic agent that prevents the degradation of HIF-1α, led to further increases in HIF-1α levels, as expected. Remarkably, CoCl_2_-induced HIF-1α upregulation was blunted by cotreatment with the C3aRA (Figure 2, D and E; compare C3aRA with C3aRA-CoCl_2_). This result was further validated by incubating WT and C3aR-KO microglial cultures under normoxic or hypoxic conditions, showing elevated HIF-1α immunostaining (Figure 2, F and G) and *Hif1a* mRNA expression (Figure 2H) under hypoxia in WT but not C3aR-KO cultures. Quantitative PCR (qPCR) analysis of mRNA levels of HIF-1α downstream targets showed that treating the WT microglial cells with CoCl_2_ resulted in increased expression of glycolysis-promoting targets *Pdk1* (Figure 2I), *Pfkl* (Figure 2J), and *Pfk2* (Figure 2K). Consistent with HIF-1α expression dampened by *C3ar1* deficiency, the degree of induction of these HIF-1α targets was significantly lower in CoCl_2_-treated C3aR-KO microglia compared with WT cells (Figure 2, I–K, and Supplemental Table 3).

To assess the overall effect of the C3aR/HIF-1α axis on microglial metabolic fitness, we performed Seahorse assays in WT and C3aR-KO primary microglial cultures (Figure 2L). We found that, while basal responses were similar between WT and C3aR-KO (Figure 2, M–O; compare WT-vehicle with C3aR-KO–vehicle), treating the WT cultures with CoCl_2_ led to a dramatic reduction in both the basal oxygen consumption rate (OCR) and maximal OCR measured after injection of FCCP, a potent uncoupler of mitochondrial OXPHOS (Figure 2, M and N; compare WT-vehicle with WT-CoCl_2_) as well as ATP production OCR (Figure 2O). Strikingly, the C3aR-KO cultures were resistant to CoCl_2_ treatment, displaying similar basal respiration, maximal respiration, and ATP production OCR compared with vehicle-treated WT and C3aR-KO cells (Figure 2, M–O).

**Figure 3:**
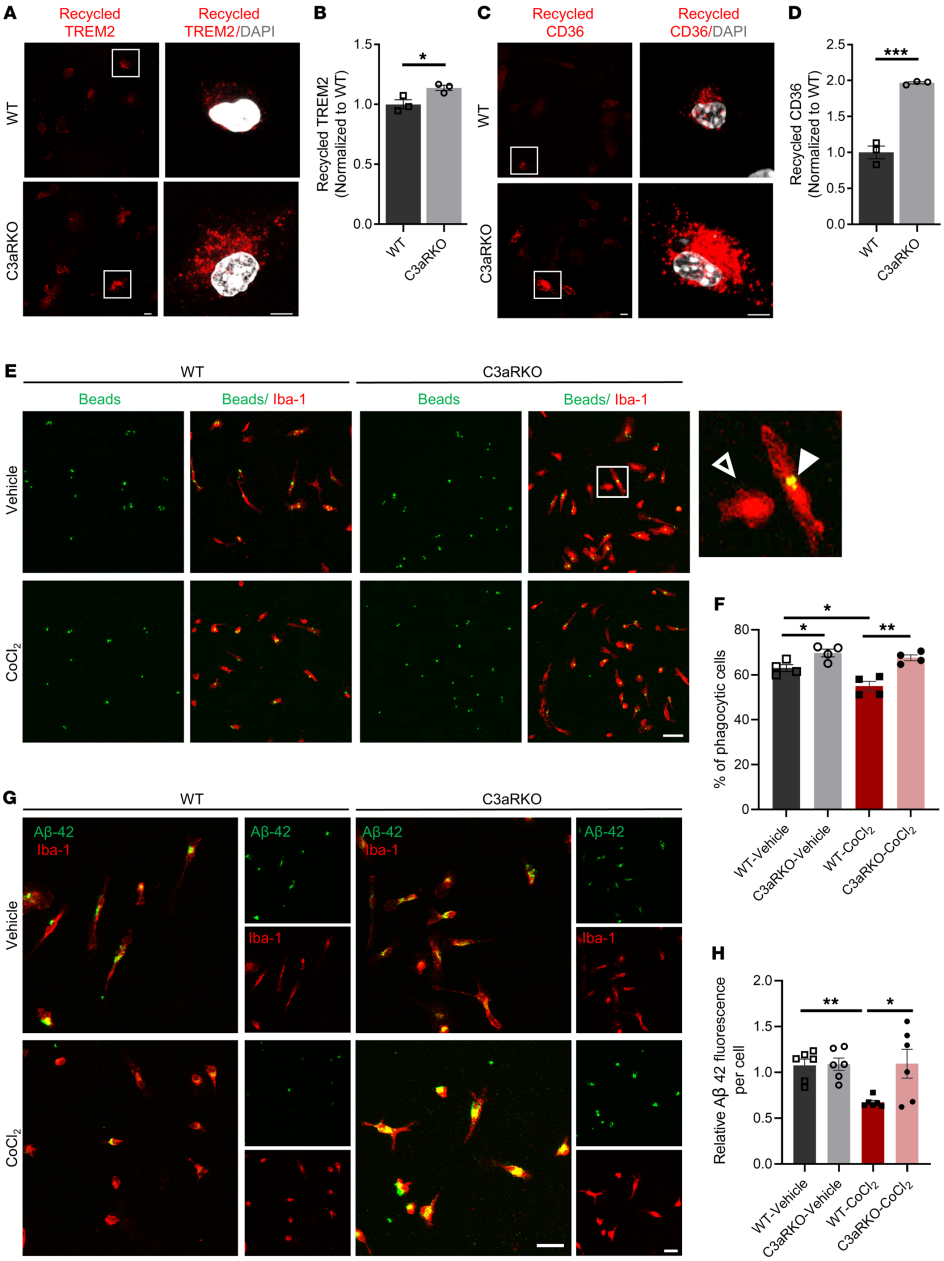
C3aR-depleted microglia are resistant to hypoxia-induced phagocytic changes. (**A**) Representative images of recycled TREM2 (red) and DAPI (gray) in WT and C3aR-KO microglia. Scale bars: 10 μm. (**B**) Quantification of TREM2 recycling. (**C**) Representative images of recycled CD36 (red) and DAPI (gray) in WT and C3aR-KO microglia. Scale bars: 10 μm. (**D**) Quantification of CD36 recycling. (**E**) Representative images for bead uptake (green) and Iba-1 (red) in WT and C3aR-KO microglial cells treated with 100 μM CoCl_2_ for 48 hours. Zoomed-in images show phagocytic (filled white arrowhead) and nonphagocytic (hollow white arrowhead) cells. Scale bar: 50 μm. (**F**) Quantification of percentage of phagocytic cells. (**G**) Representative images from Aβ42 uptake (green) and Iba-1 (red) in WT and C3aR-KO microglial cells treated with 100 μM CoCl_2_ for 48 hours. Scale bars: 100 μm. (**H**) Quantification of percentage of phagocytic cells in **G**. For all panels, data are presented as mean ± SEM. *P < 0.05; **P < 0.01; ***P < 0.001 by 2-sided Student’s *t* test (**B** and **D**) or 1-way ANOVA with Tukey’s correction (**F** and **H**). Each experiment was repeated 2–3 times.

Having established a beneficial effect of C3aR deletion on microglial metabolic fitness, we went on to test its functional effect on phagocytosis. We first performed a receptor recycling assay, as microglial phagocytosis depends partially on their ability to recycle cell surface receptors. We found that microglial cells with depleted C3aR had higher recycling of TREM2 and CD36, which are well-recognized phagocytic receptors for Aβ, compared with WT microglia (Figure 3, A–D). Similar results were obtained by treating the BV-2 cells with C3aRA (Supplemental Figure 3). Consistent with this finding, measurement of bead uptake in primary microglial cells showed higher levels of internalized beads in C3aR-KO cells compared with WT controls under both basal and hypoxia mimetic–treated conditions (Figure 3, E and F). Similar analysis of Aβ42 phagocytosis showed that, although the baseline uptake was indistinguishable between WT and C3aR-KO cells, CoCl_2_ treatment resulted in a dramatic reduction in Aβ uptake in WT cells, and the degree of the reduction was significantly lower in C3aR-KO cells (Figure 3, G and H).

**Figure 4:**
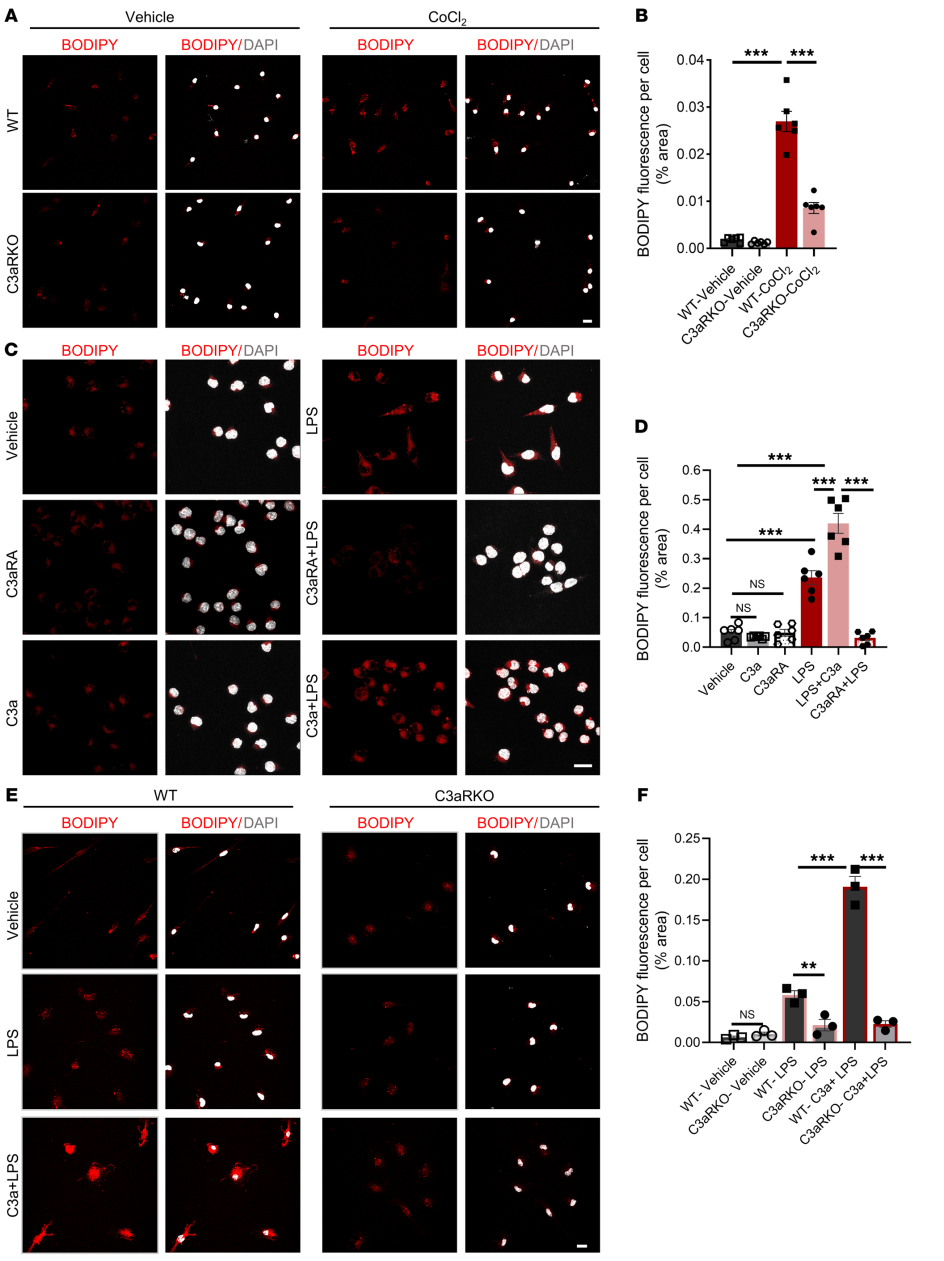
*C3ar1* deletion reduces microglial lipid droplet accumulation in vitro. (**A**) Representative images of BODIPY (red) and DAPI (gray) from 24-hour 100 μM CoCl_2_–treated WT and C3aR-KO cells. Scale bar: 15 μm. (**B**) Quantification of BODIPY^+^ area per cell from panel **A**. (**C**) Representative images of BODIPY (red) and DAPI (gray) in BV-2 cells treated with 500 nM C3a, 10 μM C3aRA, 5 μg/mL LPS, C3a + LPS, and 1-hour C3aRA pretreatment followed by LPS for 18 hours. Scale bar: 15 μm. (**D**) Quantification of BODIPY^+^ area per cell from panel **C**. (**E**) Representative images of BODIPY (red) and DAPI (gray) showing lipid droplet accumulation in WT and C3aR-KO cells after 18-hour treatment with LPS or C3a + LPS cotreatment. Scale bar: 15 μm. (**F**) Quantification of BODIPY^+^ area per cell from panel **E**. For all panels, data are presented as mean ± SEM. NS, nonsignificant. **P < 0.01; ***P < 0.001 by 1-way ANOVA with Tukey’s correction. Each experiment was repeated 2–3 times.

The effect of HIF-1α on lipid metabolism is well documented in the literature (38). Given that C3aR-positive microglia in APP-KI mice displayed a significant upregulation of genes involved in HIF-1α signaling and lipid metabolism, we wanted to evaluate the effect of C3aR on overall lipid dynamics and its relationship with the HIF-1α pathway. Measurement of BODIPY-positive lipid droplet accumulation in WT and C3aR-KO cells showed that, whereas there were no appreciable differences between the 2 groups at baseline, there was a significant accumulation of BODIPY-positive lipid droplets in WT microglia compared with C3aR-KO cells when these cells were treated with CoCl_2_ (Figure 4, A and B). Since lipid droplet accumulation in microglia is also observed in inflammatory conditions, we first treated BV-2 cells with LPS, C3aR ligand C3a, or C3aRA either separately or in combination. We found that LPS treatment led to a significant accumulation of BODIPY-positive lipid droplets (Figure 4, C and D). Although C3a or C3aRA treatment alone did not have an appreciable effect, cotreatment with LPS and C3a further augmented the lipid droplet accumulation, and these effects were abolished by pretreating the cells with C3aRA (Figure 4, C and D). Similarly, treating the primary microglial cultures with LPS led to a significant upregulation of BODIPY-positive lipid droplet accumulation in WT cells compared with C3aR-KO cells (Figure 4, E and F). Cotreatment with LPS and C3a resulted in an even greater accumulation of lipid droplets in WT microglial cells but not in C3aR-KO cultures. These results support a causal role of C3a/C3aR signaling in mediating LPS-induced lipid droplet accumulation.

Overall, the studies combined demonstrate that C3aR deficiency leads to lower HIF-1α levels and higher ATP production, resulting in resistance to hypoxia mimetic–induced metabolic and functional perturbations, as demonstrated by higher phagocytic activity and reduced lipid droplet accumulation.

### Deletion of C3ar1 reverses lipid dysregulation in APP-KI mice.

**Figure 5:**
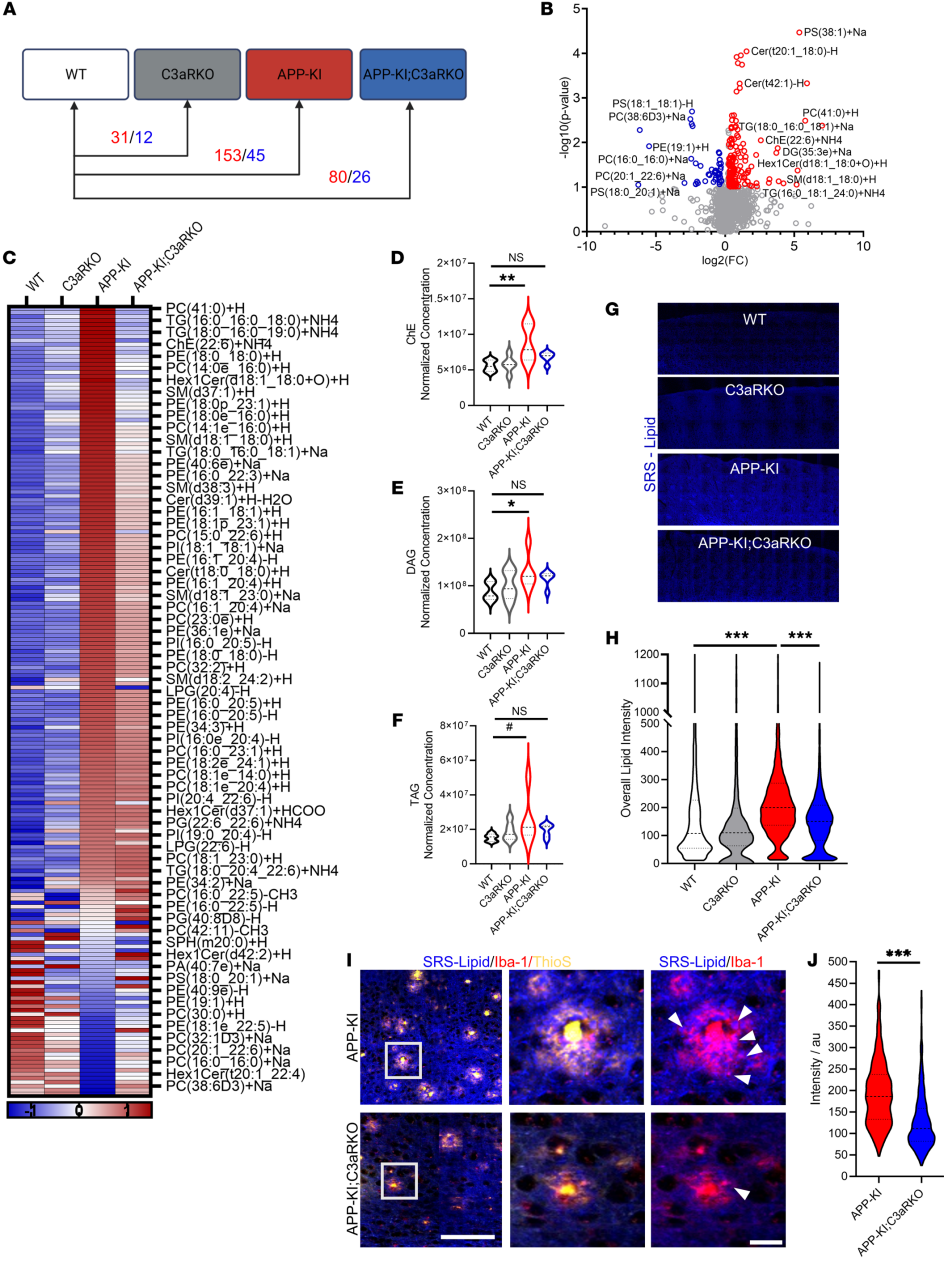
Deletion of *C3ar1* alters lipid dynamics in vivo. (**A**) Diagram showing the number of lipid species upregulated (red) or downregulated (blue) in 9-month-old C3aR-KO, APP-KI, and APP-KI; C3aR-KO mice compared with littermate WT controls. (**B**) Volcano plot showing lipid species that are upregulated (red) or downregulated (blue) in 9-month-old APP-KI compared with WT mice. (**C**) Heatmap displaying the expression of the 198 lipid species altered in APP-KI mice across all 4 genotypes. (**D**–**F**) Volcano plots of normalized lipid concentration of (**D**) cholesterol esters (ChE), (**E**) diacylglyceride (DAG), and (**F**) triacylglyceride (TAG). (**G**) Representative images from 9-month-old WT, C3aR-KO, APP-KI, and APP-KI; C3aR-KO cortical tissue showing SRS-lipid signal (blue). Imaging performed using 63× objective with overlapped tiling and stitching to cover the entire cortical region. (**H**) Quantification of overall SRS lipid intensity signal from panel **G**. (**I**) Representative images from SRS microscopy imaging performed on cortical region of 9-month-old tissues from APP-KI and APP-KI; C3aR-KO mice showing lipid^+^ (blue), Iba-1^+^ (red), and thioflavin S^+^ plaques (yellow). Arrows mark the lipid accumulation within plaque-associated Iba-1^+^ microglia. Scale bar: 20 μm. (**J**) Quantification of average lipid intensity within microglia in APP-KI and APP-KI; C3aR-KO cortical sections. For all panels, data are presented as mean ± SEM. NS, nonsignificant. ^#^*P* = 0.06; *P < 0.05; **P < 0.01; ***P < 0.001 by 1-way ANOVA with Tukey’s correction (**D**–**F** and **H**) or by 2-sided Student’s *t* test (**J**).

To investigate the effect of C3aR in lipid regulation and AD pathogenesis in vivo, we crossed the APP-KI mice with the C3aR-KO mice (Supplemental Figure 4). RNA-seq of sorted microglia from 9-month-old WT, C3aR-KO, APP-KI, and APP-KI; C3aR-KO mice followed by gene set enrichment analysis (GSEA) showed that deletion of *C3ar1* in APP-KI mice resulted in partial rescue of genes that were upregulated in APP-KI mice (Supplemental Figure 5A), particularly in pathways associated with AD, inflammation, and immune system activation. Interestingly, pathways associated with metabolism such as type I diabetes mellitus and glycerolipid metabolism were found to be upregulated in APP-KI microglia and rescued in APP-KI; C3aR-KO microglia (Supplemental Figure 5B). We next performed untargeted lipidomic analysis using mass spectrometry on 9-month-old cortical tissues from WT, C3aR-KO, APP-KI, and APP-KI; C3aR-KO mice. Partial least square discriminant analysis (PLS-DA) of the 2128 lipid species demonstrated a clear separation between WT and APP-KI, with C3aR-KO and APP-KI; C3aR-KO in between (Supplemental Figure 6A). Genotype comparisons of overall lipid species revealed that, compared with WT, the APP-KI mice exhibited 153 upregulated and 45 downregulated species, while APP-KI; C3aR-KO yielded 80 upregulated and 26 downregulated lipid species (fold change >20% and *P* value threshold <10% by 2-tailed *t* test) (Figure 5A). Examination of the 198 lipid species altered in APP-KI (Figure 5, B and C, and Supplemental Table 4) and comparison with APP-KI; C3aR-KO revealed an overall dampened signature in absence of C3aR (Figure 5C and Supplemental Table 5).

Characterization of the different lipid classes showed that those contributing to lipid droplet formation, such as sterols and glycerolipids, were enriched in APP-KI mice but not in APP-KI; C3aR-KO compared with WT (Supplemental Figure 6, B and C). Additionally, lipids from these classes were also found to be downregulated in APP-KI; C3aR-KO compared with APP-KI (Supplemental Figure 6D), indicating reduced lipid droplet–forming lipids resulting from *C3ar1* deficiency. Indeed, further assessment of normalized expression of lipid species within these classes identified significant increases in cholesterol esters (ChEs) and diacylglycerides (DAGs), with triacylglycerides (TAGs) showing a similar trend, in APP-KI compared with WT, but no appreciable differences were found between WT and APP-KI; C3aR-KO samples (Figure 5, D–F).

We next performed label-free imaging using a stimulated Raman scattering (SRS) microscopy to visualize lipid changes in intact mouse tissues of 9-month-old WT, C3aR-KO, APP-KI, and APP-KI; C3aR-KO mice (Figure 5G). We found a significant increase in lipid signals in APP-KI mice compared to WT, which was normalized in APP-KI; C3aR-KO (Figure 5H). Further examination of the lipids in microglia surrounding Aβ plaques by overlaying the SRS signal with Iba-1 and thioflavin S (ThioS) staining revealed significant downregulation in APP-KI; C3aR-KO compared with littermate APP-KI controls (Figure 5, I and J). This result was supported by reduced lipid droplet surface protein perilipin 2 (Plin2) expression in the microglia of APP-KI; C3aR-KO mice compared with that of APP-KI (Supplemental Figure 6E).

Together, our analysis identified significant alterations in lipid metabolism in APP-KI mice, with upregulation of lipid classes contributing to lipid droplets. These phenotypes were partially alleviated by *C3ar1* deficiency.

### Deletion of C3ar1 reduces HIF-1α accumulation and improves microglial barrier function and neuronal integrity.

**Figure 6:**
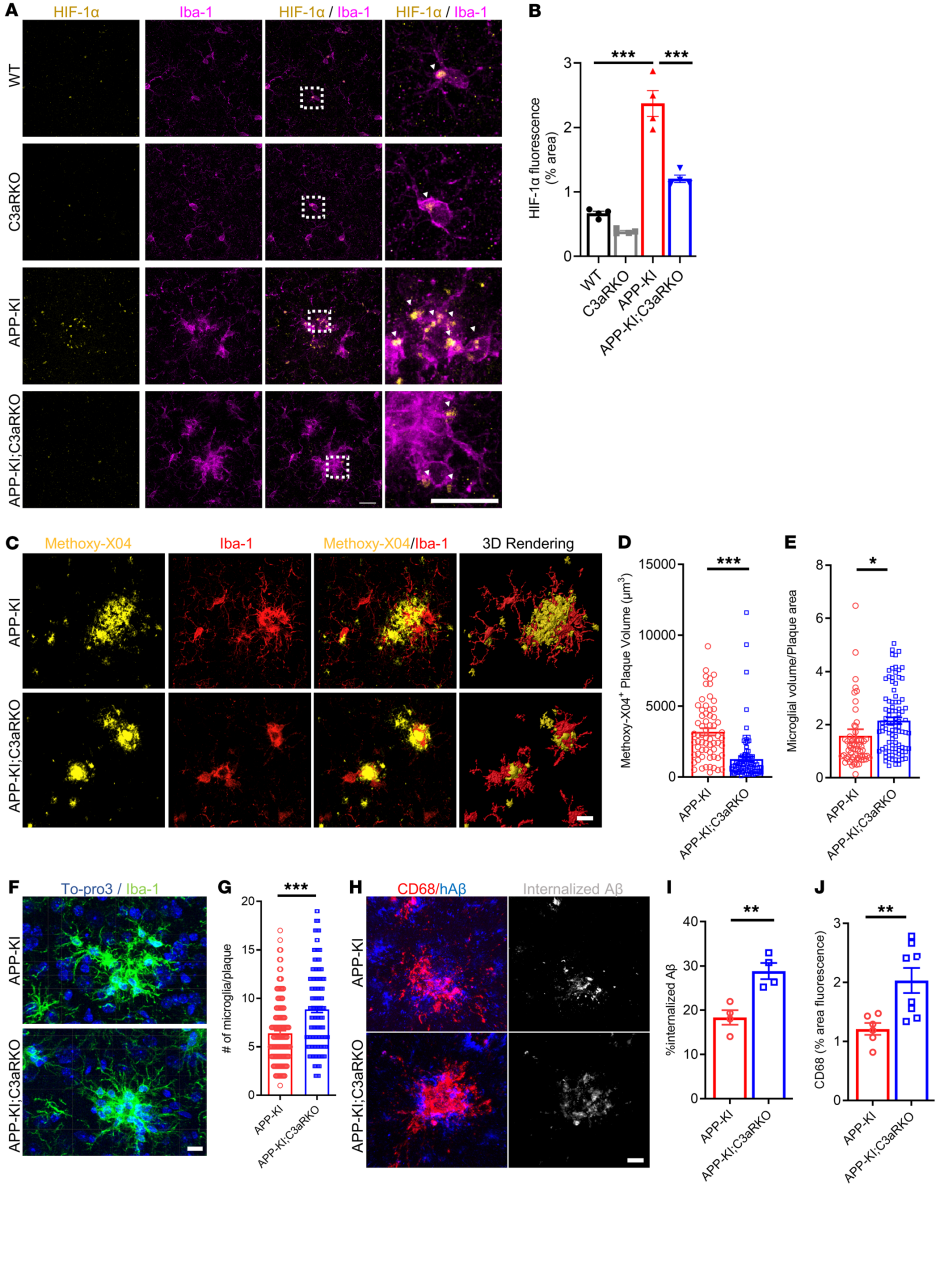
Deletion of *C3ar1* improves microglia-plaque interaction and improves microglial phagocytosis. (**A**) Representative immunofluorescence images of HIF-1α (yellow) in Iba-1^+^ microglia (magenta) across 4 genotypes. Scale bar: 15 μm. (**B**) Quantification of percentage area of HIF-1α fluorescence in WT, C3aR-KO, APP-KI, and APP-KI; C3aR-KO cortical sections. (**C**) Representative images of methoxy XO-4 (yellow) and Iba-1 (red) and 3D surface reconstruction from APP-KI and APP-KI; C3aR-KO cortical tissue. Scale bar: 15 μm. (**D**) Quantification of methoxy-X04^+^ volume in APP-KI and APP-KI; C3aR-KO mice. (**E**) Ratio of plaque-associated microglia volume/plaque area. (**F**) Representative images of To-pro3 (blue) and Iba-1 (green) in APP-KI and APP-KI; C3aR-KO mice. Scale bar: 15 μm. (**G**) Quantification of number of microglia per plaque from **F**. (**H**) Representative images of CD68 (red) and human Aβ (hAβ, blue) expression in APP-KI and APP-KI; C3aR-KO mice. The last panel shows internalized Aβ (gray) quantified by colocalization of CD68 and hAβ. Scale bar: 15 μm. (**I**) Quantification of CD68 expression from **H** (*n* = 7–8 mice/group). (**J**) Quantification of internalized Aβ from **H** (*n =* 80–100 plaques from 4 mice/group). For all panels, data are presented as mean ± SEM. *P < 0.05; **P < 0.01; ***P < 0.001 by 2-sided Student’s *t* test (**B**) or 1-way ANOVA with Tukey’s correction (**D**, **E**, **G**, **I**, and **J**).

Our in vitro data established a C3aR/HIF-1α axis that modulates microglia metabolic capacity and phagocytosis. To test the in vivo relevance, we performed immunofluorescent staining of sections from 9-month-old WT, C3aR-KO, APP-KI, and APP-KI; C3aR-KO mice with an anti–HIF-1α antibody (Figure 6A), which showed that, in agreement with our transcriptomic data, there was significantly higher HIF-1α intensity in microglia of APP-KI mice compared with WT and C3aR-KO controls, while C3aR ablation led to significantly reduced HIF-1α expression (Figure 6, A and B). This was associated with tighter plaque compaction and higher microglia clustering surrounding methoxy-X04^+^ plaques (Figure 6C), quantified by reduced methoxy-X04^+^ plaque volume (Figure 6D) and higher microglia volume per plaque area (Figure 6E) in APP-KI; C3aR-KO compared with APP-KI mice. Further assessment of the number of microglia surrounding the plaques by Iba-1 and To-pro3 double staining showed that the number of microglia per plaque was also increased in APP-KI; C3aR-KO compared with APP-KI mice (Figure 6, F and G), which contributed to the increased microglia volume per plaque area.

Examination of microglia near Aβ deposits under higher magnification showed that, compared with the APP-KI microglia, APP-KI; C3aR-KO microglia displayed reduced cell volume as well as branch numbers and lengths (Supplemental Figure 7, A–E), indicating that microglia lacking C3aR are more phagocytic. Consistent with this notion, coimmunostaining of APP-KI and APP-KI; C3aR-KO brain sections for CD68, a phagocytic microglia marker, and Aβ showed that microglia from the APP-KI; C3aR-KO mice had higher CD68 expression and these CD68^+^ phagosomes contained more internalized Aβ compared with those of the APP-KI mice (Figure 6, H–J). Increased phagocytosis was also supported by qPCR analysis, which revealed higher expression of phagocytic markers in APP-KI; C3aR-KO mice compared with the APP-KI mice (Supplemental Figure 7F and Supplemental Table 3). In contrast to CD68, expression of Clec7a, a marker for neurodegenerative and reactive microglia, was found to be significantly reduced in APP-KI; C3aR-KO mice compared with APP-KI mice (Supplemental Figure 8, A and B), indicating that the increased CD68 intensity was not a result of an overall increase in microglia reactivity. This was corroborated by reduced GFAP immunoreactivity in APP-KI; C3aR-KO compared with APP-KI mice, indicating dampened reactive astrogliosis resulting from C3aR inactivation (Supplemental Figure 8, C–E). Similarly increased CD68 and reduced Clec7a levels due to C3aR inactivation were also observed in 6-month-old mice (Supplemental Figure 9).

**Figure 7:**
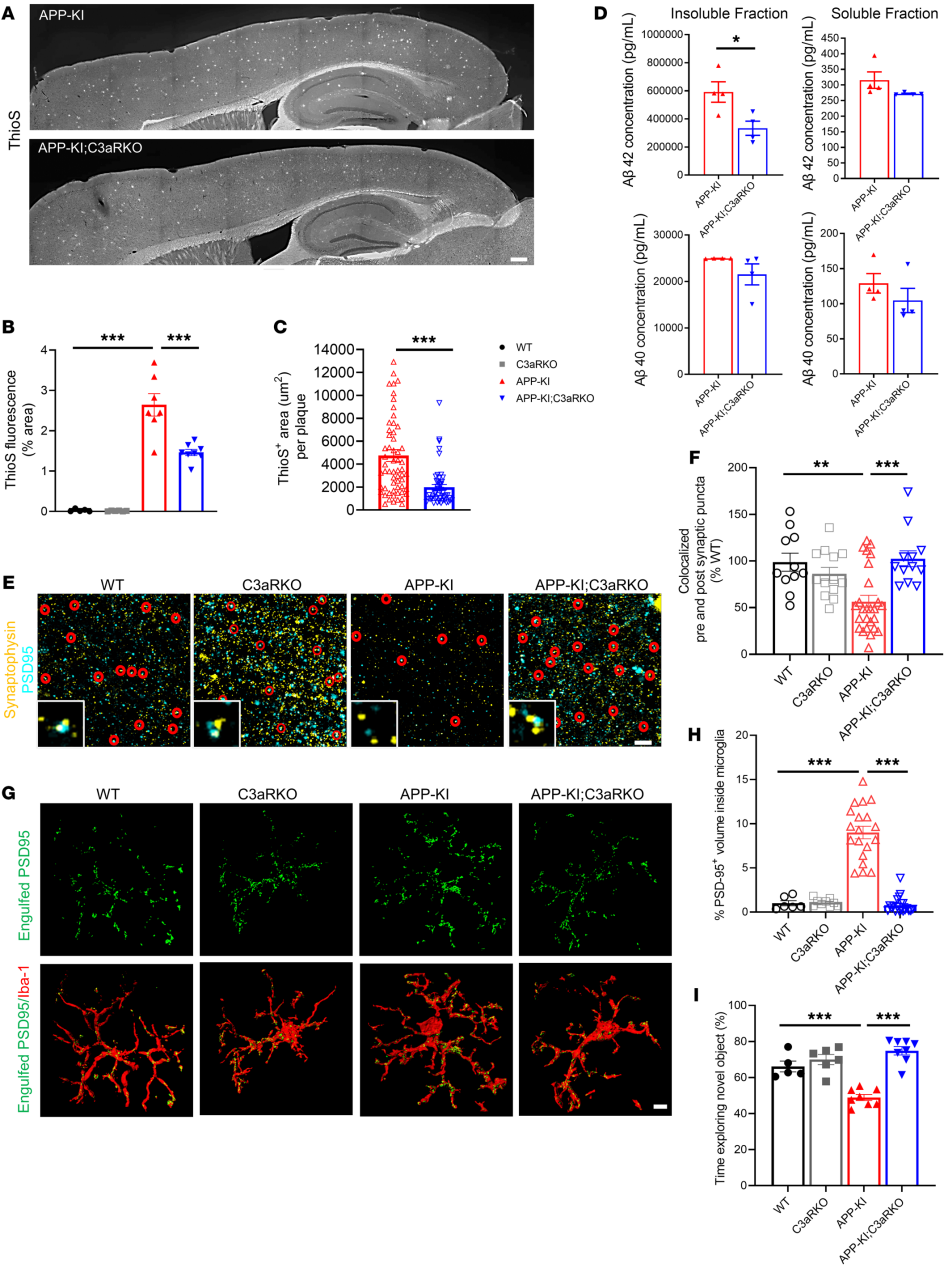
Absence of C3aR reduces Aβ pathology and improves cognitive deficits. (**A**) Representative microscopy images of thioflavin S (ThioS) from 9-month-old APP-KI and APP-KI; C3aR-KO mice. Scale bar: 1 mm. (**B**) Quantification of percentage total ThioS^+^ area in 9-month-old animals *(n* = 7–8 mice/group*)*. (**C**) Quantification of total size of ThioS^+^ plaques from APP-KI and APP-KI; C3aR-KO mice. (**D**) Results from ELISA measurement of Aβ42 and Aβ40 concentrations in guanidine hydrochloride fraction (insoluble) and TBS fraction (soluble) from 9-month-old APP-KI and APP-KI; C3aR-KO animals (*n* = 4 mice/group). (**E**) Representative images of presynaptic marker synaptophysin (yellow) and postsynaptic marker PSD-95 (cyan) from 9-month-old animals. Inset showing enlarged view of colocalization of synaptophysin and PSD-95. (**F**) Quantification of colocalized synaptophysin and PSD-95 puncta from **E** (*n =* 6 mice/group). Scale bar: 5 μm. (**G**) Representative 3D reconstruction and rendering of PSD-95 (green) signal inside Iba-1^+^ microglia (red) from 9-month-old animals *(n =* 6 mice/group*)*. Scale bar: 5 μm. (**H**) Quantification of PSD-95 volume inside microglia. (**I**) Results from novel object recognition assay of 9-month-old WT, C3aR-KO, APP-KI, and APP-KI; C3aR-KO animals (*n =* 8-10 mice/group). For all panels, data are presented as mean ± SEM. *P < 0.05; **P < 0.01; ***P < 0.001 by 1-way ANOVA with Tukey’s correction (**B**, **F**, **H**, and **I**) or 2-sided Student’s *t* test (**C** and **D**).

The above results demonstrate that absence of C3aR leads to reduced HIF-1α expression in microglia surrounding Aβ plaques and increased microglial barrier function and phagocytosis. To understand how the C3aR-mediated changes in microglia dynamics affect overall Aβ pathology and functional outcomes, we performed ThioS staining in 9-month-old mouse tissues of APP-KI and APP-KI; C3aR-KO mice. We found a significant reduction in ThioS-positive area as well as the size of ThioS-positive plaques in APP-KI; C3aR-KO compared with APP-KI mice (Figure 7, A–C). This was also the case when the mice were analyzed at 6 months of age (Supplemental Figure 9, A and C). Reduced Aβ plaque pathology resulting from C3aR deficiency was corroborated by ELISA analysis of Aβ40 and Aβ42 species in Tris-buffered saline (TBS) and guanidine hydrochloride fractions from 9-month-old cortical tissues of APP-KI and APP-KI; C3aR-KO mice, showing a significant reduction in Aβ42 concentration in the guanidine hydrochloride fraction in APP-KI; C3aR-KO samples compared with APP-KI, but not the TBS-soluble fractions (Figure 7D). Thus, C3aR depletion had a major effect in reducing insoluble Aβ42, likely through improved phagocytic function.

Next, we evaluated the functional effect of C3aR KO on synaptic health and cognition. We stained brain sections from 9-month-old WT, C3aR-KO, APP-KI, and APP-KI; C3aR-KO mice for presynaptic marker synaptophysin and postsynaptic marker PSD-95 (Figure 7E). We found a significant reduction in synaptophysin and PSD-95 immunoreactivities (Supplemental Figure 10, A and B) as well as colocalization of synaptophysin and PSD-95 puncta (Figure 7F) in APP-KI mice compared with WT and C3aR-KO controls. The degree of colocalization in APP-KI; C3aR-KO mice was comparable to WT and C3aR-KO samples, demonstrating a rescue of synapse loss in APP-KI mice by C3aR ablation (Figure 7F). The synaptic marker levels were inversely correlated with the synapses engulfed by microglia quantified by the volume of PSD-95–positive puncta inside microglia, which revealed a significant increase in APP-KI mice compared with WT and C3aR-KO groups, and restoration to control levels in APP-KI; C3aR-KO mice (Figure 7, G and H). Overall, these results are consistent with an earlier report demonstrating a protective role of C3aR deficiency against synapse loss (39).

Assessment of LAMP-1^+^ dystrophic neurites surrounding the plaques also showed reduced LAMP-1 expression in APP-KI; C3aR-KO mice compared with APP-KI mice (Supplemental Figure 10, C and D). With the rescue of synapses and reduction in dystrophic neurites, we wanted to test the effect of C3aR on overall behavioral changes. We performed open field assessment on 9-month-old WT, C3aR-KO, APP-KI, and APP-KI; C3aR-KO mice and found no significant differences (Supplemental Figure 11, A–C), indicating that C3aR did not affect anxiety or mobility in these mice. To investigate the impact of C3aR on memory and learning, we chose to perform novel object recognition behavioral assays on 9-month-old mice, based on previous studies demonstrating deficits in APP-KI mice using this test (40, 41). We found that the APP-KI mice had significantly reduced exploration time with the novel object compared with the WT and C3aR-KO mice, and this phenotype was rescued by C3aR deficiency (Figure 7I). Behavioral analysis of fear conditioning did not reveal genotype differences, in line with previous literature (40, 42). (Supplemental Figure 11, D–H). Overall, these results demonstrate that absence of C3aR rescued the synapse loss and behavioral deficits observed in APP-KI mice.

## Discussion

In AD, microglia cluster around the Aβ deposits, forming a protective barrier and facilitating Aβ phagocytosis and clearance. This process is energy intensive, necessitating sufficient supply of ATP (43). The microglial barrier and phagocytic functions can be altered by genetic modifiers and by the local brain environment. For example, loss of triggering receptor expressed in myeloid cells (*TREM2*), a late-onset-AD gene, is associated with impaired microglial metabolism and clustering and these phenotypes can be rescued by boosting ATP levels (4). Likewise, elevated levels of hexokinase 2 (HK2), a crucial enzyme in regulating glucose metabolism and glycolysis, has been detected in microglia proximal to Aβ plaques. Ablation of HK2 results in increased ATP production and improved microglial response to Aβ (6). Here we reveal a complement C3aR and HIF-1α signaling pathway that regulates microglial metabolism and ATP production in vitro and its barrier and phagocytic function in APP-KI mice. These studies combined support a model whereby membrane receptors such as C3aR and TREM2 respond to Aβ to converge on intracellular signaling pathways, with shared activities in regulating microglial metabolism and energy status, which in turn confer their barrier and phagocytic capacities.

We find that C3aR is prominently expressed in microglia proximal to Aβ plaques and these C3aR-positive microglia share many gene signatures with disease-associated microglia, with upregulation of reactive microglial genes and downregulation of microglia homeostatic genes (35, 37, 44). Intriguingly, we identified a dysregulated metabolic signature in these C3aR-positive microglia, characterized by an activated HIF-1α signaling pathway along with upregulation of genes and pathways involved in lipid metabolism. Similarly compromised hypoxia and lipid metabolic profiles have recently been reported in Aβ-associated microglia in other AD models (16, 45, 46). The hypoxia module was not a prominent feature in other neurodegenerative conditions such as multiple sclerosis or amyotrophic lateral sclerosis (35), suggesting that these metabolic signatures are uniquely Aβ induced.

Consistent with our RNA-seq data, we detected significant upregulation of HIF-1α mRNA and protein expression in microglia surrounding the plaques along with increased expression of its downstream targets. Sustained hypoxia or overstabilization of HIF-1 has been shown to result in microglial quiescence and reduced Aβ clustering, leading to increased plaque pathology (16). These phenotypes are reminiscent of TREM2 deficiency (47–49), indicating a possible interaction between HIF-1α and TREM2. Our results lend further support for this crosstalk. Specifically, C3aR deficiency results in reduced HIF-1α expression and resistance to hypoxia-induced metabolic impairment. This is associated with increased expression of TREM2 and its adapter TYROBP (also known as DAP12) and enhanced TREM2 recycling and its cell-surface levels. However, this is likely due to a general effect of the C3aR/HIF-1α axis in microglial metabolism and cell surface receptor recycling rather than a specific role of this pathway in TREM2 regulation. In addition, while we place TREM2 as a downstream factor of the C3aR/HIF-1α signaling, TREM2 is also known to play a pivotal role in maintaining metabolic fitness of myeloid cells. Absence of TREM2 leads to compromised metabolism and reduced ATP production, similar to HIF-1 overactivation. In this regard, it is conceivable that TREM2 may influence the C3aR/HIF-1α pathway through its role in cellular metabolism. The exact relationship between C3aR, HIF-1α, and TREM2 warrants further investigation.

Although our current studies focus on examining the intracellular pathways downstream of C3aR rather than ligand-receptor interaction, it is worth noting that apart from it bona fide ligand C3a, C3aR also interacts with TLQP-21, a 21–amino acid peptide derived from pro-peptide VGF (50). The interplay between C3a and TLQP-21 in C3aR/HIF-1α regulation is an intriguing question but remains to be elucidated. Also of interest is a recent study linking aberrant *S*-nitrosylation of C3 to AD in women (51). Whether different forms of C3/C3a elicit distinct C3aR interaction and downstream effect warrants further study. We did not observe overt sex-specific differences with regard to Aβ pathology or behavior in our models for the ages we analyzed, which is in line with other reports in the literature (34). Nevertheless, in light of the new report, a possible sex-specific effect of the C3aR and HIF-1α interaction is a valid question that deserves more detailed analysis.

Phagocytosis, a major function of the microglia to maintain brain homeostasis, is compromised with age and in pathological conditions such as AD (52). Impairments in expression, recycling, and trafficking of phagocytic receptors such as TREM2 and CD36 contribute to neurodegeneration (4, 53, 54), while their upregulation results in improved phagocytosis and decelerated AD pathogenesis (55–58). Besides their roles in Aβ phagocytosis, TREM2 and CD36 are also involved in lipid sensing, whereas the HIF-1α pathway mediates lipid uptake and storage (38). Consistent with this notion, we show that genetic ablation of C3aR leads to increased trafficking and recycling of TREM2 and CD36, elevated Aβ phagocytosis, and reduced lipid droplet accumulation induced by hypoxia mimetic treatment. This is associated with higher CD68 immunoreactivity and reduced lipid droplets visualized by SRS microscopy in APP-KI mice with C3aR deficiency. Interestingly, untargeted lipidomic analysis of mouse cortical tissues identified profound changes in lipid species in APP-KI mice compared with WT controls, and these were partially rescued by C3aR ablation. How disruption of a predominantly microglial pathway leads to overall rescue of the lipid profile is an interesting question requiring further investigation. Besides TREM2 and CD36, other phagocytic receptors are likely affected by the C3aR/HIF-1α pathway and may contribute to the overall effects. Indeed, our qPCR analysis revealed changes in multiple molecules involved in microglial phagocytosis in C3aR-KO cells.

Here, we identify a C3aR/HIF-1α axis mediating microglial metabolism and function. Disrupting this axis by C3aR ablation results in increased energy levels and resistance to hypoxia-induced metabolic changes, leading to improved microglial phagocytosis and barrier formation and reduced Aβ pathology and improved cognition. Our study places microglial metabolism and ATP as crucial mediators of microglial function, which are shared with other AD-associated pathways, particularly TREM2. Of note, the metabolic and phagocytic deficits caused by TREM2 deficiency can also be reversed by cyclocreatine, an ATP analog (4), and by treatment with sodium rutin, a flavonoid that induces a switch from glycolysis to OXPHOS (3), raising the possibility that boosting microglial metabolic and energy homeostasis may be a potential therapeutic approach for AD treatment. Our work provides support to the idea that this may also be accomplished by C3aR blockade or HIF-1α inhibition, both of which are therapeutically actionable.

## Methods

### Mice

C3aR-KO mice were obtained from the Jackson Laboratory and backcrossed with C57BL/6J (Jackson Laboratory) for 5 generations prior to crossing with *APP*^NL-G-F^–knockin (APP-KI) mice (RIKEN) to obtain littermate WT, C3aR-KO, APP-KI, and APP-KI; C3aR-KO. Homozygous *APP*^NL-G-F^ were crossed with a homozygous Td-tomato reporter *C3ar1^fl/fl^* line to generate heterozygous APP-KI; Td-tomato breeding pairs, which were crossed to obtain littermate WT; Td-tomato and APP-KI; Td-tomato mice. Mice were housed 2–4 per cage in a pathogen-free mouse facility with ad libitum access to food and water on a 12-hour light/12-hour dark cycle. Male and female mice were used in approximately equal numbers. Sex-specific differences were not found at the ages analyzed in this study. All analyses were performed on 9-month-old animals unless mentioned otherwise.

### Human AD brain specimens

Postmortem brain tissues were provided by the University of Pennsylvania Center for Neurodegenerative Disease Research (CNDR). Tissues were embedded in paraffin, sectioned at a thickness of 7 μm, and were stained as described in Histology and immunofluorescence below.

### FACS-based isolation of microglia from adult mouse brain

FACS-based isolation of microglia was performed as previously described, with minor modifications (59). Briefly, adult mice were perfused with PBS, dissected brain tissues were gently minced with sterile razor blades, digested in papain (LK003172, Worthington Biochemical) and DNase (LK003178, Worthington Biochemical), and triturated 5–6 times using a sterile fire-polished glass Pasteur pipette. After incubation, papain digestion was neutralized by adding ice-cold HBSS+ (HBSS with 2 mM EDTA and 0.5% BSA) and the suspension was pelleted at 310*g* for 5 minutes at 4°C. The pellet was resuspended in 1 mL of HBSS+, transferred to an ice-cold 1.7 mL Eppendorf tube, further triturated 5–6 times, and the supernatant was collected after a brief, low-speed centrifugation. After each brief centrifugation, the supernatant was filtered through a previously wetted 40 μm cell strainer (352340, BD Biosciences) into a chilled 50 mL conical tube and centrifuged at 310*g* for 5 minutes at 4°C. The resulting pellet was resuspended in 20% 4°C Percoll PLUS (E0414, MilliporeSigma) in 1× PBS and centrifuged with low brake at 310*g* at 4°C for 20 minutes. The resulting pellet containing single cells was incubated in 500 μL HBSS+ containing Mouse BD Fc Block (1:100; 553141, BD Biosciences). Antibodies were rat anti-CD45–BV421 (1:500; 563890, BD Biosciences) and rat anti-CD11b–FITC (1:500; 553310, BD Biosciences). The microglial population was gated and sorted based on CD45^mid^ and CD11b^+^ expression. For the C3aR expression–dependent sorting, these CD45^mid^ and CD11b^+^ cells were sorted based on Td-tomato expression. Sorting was performed using a BD Biosciences FACSAria II with a 100 μm nozzle. Cells were sorted into 1.7 mL Eppendorf tubes coated with 200 μL HBSS+, followed by lysis of pellets in Qiagen RLT buffer containing 1% β-mercaptoethanol for downstream RNA analysis.

### Primary microglial culture

Primary cultures were prepared as described previously (29). Briefly, newborn pups between P0 and P2 were used to generate primary cultures. Brain from the pups was dissected in HBSS with 10 mM HEPES, 0.6% glucose, 1% v/v Pen/Strep, and cut into small pieces. This was followed by digestion in 2.5% trypsin at 37°C for 15 minutes before addition of 1 mg/mL trypsin inhibitor. Tissue was centrifuged for 5 minutes at 400*g*, triturated, and resuspended in DMEM with 10% FBS. Cells were plated onto poly-D-lysine–coated (PDL-coated) T-75 flasks to generate mixed glial cultures. These mixed glia cultures were allowed to grow for 2 weeks, with refeeding every other day. For primary microglial culture, mouse microglial cells from mixed glial cell culture were selected with CD11b microbeads according to the manufacturer’s instructions (130-093-634, Miltenyi Biotec). Enriched microglia were plated in 24-well PDL-coated plates in DMEM with 10% FBS and 1% Pen/Strep and supplemented with 10 ng/mL M-CSF. Microglial cells were allowed to rest for 24–48 hours before treatments and RNA extraction. The experiments were repeated 2–3 times, with 3 replicates per condition.

### Phagocytic receptor recycling

Receptor recycling assays were performed as previously described (3, 53). Briefly, primary microglia were plated on PDL-coated coverslips in 24-well plates at a density of 20,000 cells per well. Cells were maintained in DMEM with 10% FBS and 1% Pen/Strep and supplemented with 10 ng/mL M-CSF for 24 hours. Cells were then incubated in DMEM with 10% donkey serum (S30-M, Sigma-Aldrich) for 15 minutes at 37°C. Antibodies against CD36 (ab23680, Abcam) or TREM2 (AF1729, R&D Systems) were added to the cells in DMEM with 1% donkey serum for 1 hour at 37°C. Cells were then acid washed with cold DMEM at pH 2.0. Cells were cultured in DMEM with 10% donkey serum for 1 hour at 37°C and then incubated with fluorophore-conjugated secondary antibodies (Alexa Fluor 555 or Alexa Fluor 647, Invitrogen) in 1% donkey serum for 1 hour at 37°C. Cells were again acid washed with cold DMEM at pH 2.0 and washed with cold PBS. Cells were then fixed with 4% paraformaldehyde (PFA), washed with PBS, and mounted on glass slides. Similar experiments were performed on BV-2 cells plated on glass coverslips and pretreated with vehicle or 10 μM C3aRA (SB290157, Calbiochem). Fluorescent signal from vesicles containing recycled receptors were thresholded and the intensity of the fluorescence signal was determined by ImageJ (NIH) and normalized to WT or vehicle-treated cells.

### Beads and Aβ uptake

Fluorescently labeled latex beads of 1 μm diameter were preopsonized in FBS for 1 hour at 37°C before dilution in cell culture medium. WT and C3aR-KO cells were seeded on PDL-coated coverslips in 24-well plates at 50,000 cells per well and allowed to rest for 24 hours prior to treatment with 100 μM CoCl_2_ (C2911, Sigma-Aldrich) for 48 hours. The treatment was washed off and the medium was replaced with bead-containing medium, and the culture was incubated at 37°C for 1 hour. Cells were washed with PBS and fixed following by immunostaining for Iba-1. FAM-labeled Aβ (AS23526-01, Anaspec) was prepared as previously described (23). Briefly, lyophilized peptide was dissolved in hexafluoroisopropanol and dried using a SpeedVac and redissolved in DMSO prior to aliquoting and freezing. FAM-labeled Aβ was incubated in complete media at 37°C for 1 hour. For each experiment, vehicle- and 100 μM CoCl_2_–treated microglial cells were treated with 100 nM Aβ42 for 24 hours, followed by washing using 1× PBS and fixing cells using 4% PFA and staining for Iba-1.

### Lipid droplet measurement

For LPS-induced lipid droplet accumulation, primary microglial cells and BV-2 cells were exposed to 5 μg/mL LPS or a combination of 5 μg/mL LPS and 500 nM C3a (8085C3-025, R&D Systems) for 18 hours. After treatment, the cells were washed with 1× PBS and fixed using 4% PFA followed by staining using BODIPY (D3835, Invitrogen). For hypoxia mimetic–induced lipid droplet accumulation, 50,000 primary microglia were plated on PDL-coated plates and were exposed to 100 μM CoCl_2_ and vehicle treatment for 24 hours. Cells were washed with 1× PBS and fixed in 4% PFA followed by staining using BODIPY.

### ATP measurement

WT and C3aR-KO primary microglial cells were seeded on PDL-coated 12-well plates and allowed to grow for 24 hours. Cells were then washed with ice-cold PBS and lysed in 1× passive lysis buffer (E1941, Promega). The lysates were split equally into 2 aliquots. One aliquot was transferred to a white opaque 96-well plate for the measurement of the ATP concentration using an ATP determination kit (A22066, Invitrogen). The other aliquot was transferred to a clear 96-well plate for the measurement of protein concentration using the BCA assay. The final ATP level was calculated by normalizing the ATP concentration in a well to its corresponding protein concentration.

### Seahorse assay

Seahorse Mito Stress Tests were performed on an Agilent Seahorse XFp Analyzer using the Mito Stress Test Kit (103010, Agilent). Briefly, 2 days prior to the assay, CD11b^+^ microglial cells from WT and C3aR-KO primary cultures were selected and plated on PDL-coated Seahorse microplates at 40,000 cells per well in the microglial medium. Twenty-four hours after seeding, the cells were exposed to 100 μM CoCl_2_ for 24 hours. The treatment was then washed off and the cells were incubated in assay medium (DMEM, 1 mM sodium pyruvate, 2 mM glutamine, and 10 mM glucose). The OCR was measured in real time during the assay, following the sequential injection of 1.5 μM oligomycin, 1 μM FCCP, and 0.5 μM rotenone/antimycin A. Data were analyzed using Seahorse XF 96 Wave software, and the results were normalized to cell number and are presented as pmol/min for OCR.

### RNA extraction, reverse transcription, and qPCR

RNA was isolated from brain tissues and FACS-isolated cell pellets by lysing them in Qiagen RLT buffer with 1% β-mercaptoethanol and processed with an RNeasy Mini isolation kit (Qiagen) according to the manufacturer’s protocols, including DNase digestion steps. RNA (1 μg) was used to generate cDNA by reverse transcription using iScript Reverse Transcription Supermix reagent (170-8840, Bio-Rad). qRT-PCR was performed using iTaq Universal SYBR Green Supermix (172-5124, Bio-Rad) on a CFX384 Touch Real-Time PCR Detection System. The relative levels of expression were quantified and analyzed by using Bio-Rad CFX manager, using *18S* and *GAPDH* as housekeeping controls.

### RNA-seq

For RNA-seq on sorted microglial cells, RNA samples were prepared as described above for RNA extraction. Library prep and RNA-seq were performed at the GARP core at Baylor College of Medicine. RNA samples were analyzed for their RIN score prior to sequencing. A NovaSeq 6000 was used for sequencing with an SP flow cell. Raw reads were first aligned to the *Mus* *musculus* genome (UCSC mm10) using TopHat v2.0.9 (http://ccb.jhu.edu/software/tophat) with default parameters. Principal component analysis showed distinct clustering of the 2 groups. Differential gene expression analysis was carried out using the DESeq2 package in the R environment (https://www.r-project.org/). Differentially expressed genes were identified with a false-discovery rate (FDR) of 0.05%. KEGG and Gene Ontology analyses were performed in the R environment as well as in DAVID (https://david-d.ncifcrf.gov/). The RNA-seq data have been deposited to the NCBI Gene Expression Omnibus (GEO GSE228258).

### Lipidomic analysis

Brain cortices from 9-month-old WT, C3aR-KO, APP-KI, and APP-KI; C3aR-KO mice were snap frozen and ground using a bead beater. Ten milligrams of each sample was collected in 1.5 mL tubes and homogenized in 200 μL of 50 mM ammonium acetate solution. Samples were normalized based on protein concentrations from the BCA assay. Splash Lipidomix Mass Spec Standard (Avanti, 330707) was spiked (10 μL) in each sample before the extraction of lipids. Lipids were then extracted using methanol, methyl *tert*-butyl ether, and water. The extracted samples were dried in an Eppendorf Vacufuge and resuspended in 110 μL isopropanol/methanol (50:50, vol/vol). The samples were analyzed using a Vanquish UPLC and Lumos orbitrap mass spectrometer (Thermo Fisher Scientific). High-throughput analysis of lipidomic data was performed using Lipidsearch software (Thermo Fisher Scientific). The statistical analysis was done using MetaboAnalyst 5.0 (https://www.metaboanalyst.ca/MetaboAnalyst/ModuleView.xhtml).

### Histology and immunofluorescence

Human brain specimens were stained as previously described (60). Briefly, slides were deparaffinized using xylene, rehydrated through an ethanol gradient, and subjected to heat-induced epitope retrieval using sodium citrate buffer. Tissues were then permeabilized in 0.5% Triton X-100 and placed in blocking solution (5% BSA and 0.5% Triton X-100 in 1× PBS) containing diluted primary antibodies in a humidified chamber for 2 days at 4°C. Sections were then rinsed with 1× PBS-Tween, quenched with True Black for 1 minute to eliminate autofluorescence, rinsed with 1× PBS, and then placed in blocking solution with secondary antibodies in a dark chamber for 4 hours at room temperature, followed by rinsing in 1× PBS. To stain tissues for plaques, following secondary antibody incubation, tissue sections were placed in 40% ethanol solution in PBS and incubated in 10 μM methoxy-X04 dye (4920, Tocris) in 40% ethanol in PBS for 15 minutes followed by rinsing in 40% ethanol in PBS and then PBS alone. Slides were then mounted in ProLong Glass Anti-fade mountant (Thermo Fisher Scientific).

For mouse brain analysis, mice were perfused with saline, followed by fixation in 4% PFA overnight at 4°C, and then transferred into 30% sucrose solution until sectioning. Sagittal brain sections (30 μm) were cut on a sliding microtome and stored at –20°C in cryoprotectant. After washing in PBS, sections were blocked with PBS containing 0.4% Triton X-100 and 4% donkey serum for 1 hour and then incubated in blocking solution containing primary antibody overnight at 4°C. After primary antibody incubation, sections were washed in 1× PBS 3 times and stained with appropriate secondary antibodies for 1 to 2 hours and washed again before mounting.

For ThioS staining, brain sections were incubated with 0.002% ThioS in TBS for 8 minutes, followed by rinsing twice in 50% ethanol for 1 minute. Then brain sections were washed 3 times in TBS for 10 minutes each before mounting and imaging. For methoxy-X04 staining, antibody-stained brain sections were dried onto slides overnight, rehydrated in PBS for 30 seconds, placed in 40% ethanol/PBS for 30 seconds, dyed with 1 μM methoxy-X04 in 40% ethanol/PBS for 30 seconds, rinsed in 40% ethanol/PBS for 30 seconds, then in 70% ethanol/PBS for 30 seconds, in 90% ethanol/PBS for 30 seconds and dipped twice in xylene, then allowed to dry once again before mounting and imaging. All antibodies and dyes used for staining are listed in Supplemental Table 6.

### Image quantification

#### CD68 and Aβ internalization.

Sagittal sections (30 μm) were stained for Iba-1 (019-19741, Wako), CD68 (MCA1957GA, Bio-Rad), and Aβ (2454S, Cell Signaling Technology) and imaged using a Leica confocal microscope. Total CD68 area fluorescence was calculated using ImageJ. For the Aβ internalization studies, *Z*-stack images were taken at 0.7 μm step size and the Co-loc function in IMARIS (Oxford Instruments) was used to quantify the amount of internalized Aβ inside CD68^+^ phagosomes.

#### Microglial characteristics and plaque interaction.

Sagittal sections (30 μm) stained for Iba-1 (019-19741, Wako) and with methoxy-XO4 were used to stain microglia and plaques, respectively. The sections were imaged on a Leica confocal microscope using a 63× oil objective with 1.5× digital zoom. *Z*-stacks were obtained for the entire thickness of the tissue, at 1 μm step size. The surface function of IMARIS was used to create 3D rendering of both microglia and plaque channels. Microglial volume and surface area were calculated within a 30 μm radius from the center of the plaque. At least 150 plaques were analyzed for each group. Microglial morphology was quantified using the filament function in IMARIS to define the microglial processes. Approximately 1000 cells were analyzed in each group. Numbers of microglia surrounding the plaque were identified by costaining of Iba-1 with To-pro3 and methoxy-X04 to identify nuclei of microglia surrounding the plaques. The number of To-pro3–positive microglia surrounding the plaque was calculated for each plaque. At least 150–200 plaques were analyzed for each group.

#### Synaptic colocalization.

Synaptic marker colocalization was performed as previously described (60). Briefly, brain sections were stained using antibodies for synaptophysin (ab16659, Abcam) and PSD-95 (MAB1596, Millipore), marking pre- and postsynaptic terminals, respectively. Imaging was performed with a Leica confocal microscope, using a 63× oil objective with a 4.0 digital zoom. *Z*-stacks of 5 μm thickness from the middle of the tissues were obtained with a 0.2 μm step size. *Z*-stacks with pre- and postsynaptic puncta were analyzed using the Spots feature of IMARIS. Spots were generated automatically (with manual adjustment for accurate puncta representation) for each channel separately, and total numbers of spots was recorded for each channel. Eventually, spots were analyzed by the Co-localize Spots MATLAB (MathWorks) plugin. Pre- and postsynaptic puncta were defined as colocalized if their centers were within 200 nm.

#### Synaptic engulfment.

Quantification of synaptic puncta inside microglia was performed as previously described using the IMARIS software (61). Briefly, immunostained brain sections were imaged with the 63× objective oil with the 4× zoom using a Leica confocal microscope with a 0.2 μm step. Microglia and PSD-95 volumes were reconstructed using IMARIS software with the Bitplane Surface function.

### SRS microscopy

Brain tissue sections were kept in stock solution. Before imaging, 80 μL PBS was deposited on a clean slide. A section of brain tissue was transferred to the PBS and flattened. A coverslip was applied to cover the liquid and brain tissue section. Excessive liquid was removed, and the periphery was sealed with UV-cured glue.

A home-built SRS microscope was integrated with a commercial confocal microscope (62). Briefly, a pump laser–integrated optical parametric oscillator (picoEmerald, APE) serves as the laser source. The picoEmerald offers 2 wavelengths: one is fixed at 1031 nm and the other is tunable from 700 nm to 990 nm. To image lipid content, the tunable wavelength was set at 796.9 nm; therefore, the 2 wavelength’s frequency difference is in resonance with CH2 symmetric vibration. The microscope is capable of laser scanning confocal microscopy. Before imaging the tissue, calibration was performed using fluorescent polystyrene microsphere of 1 μm diameter. The microsphere was imaged with both fluorescence and SRS, followed by fine-tuning of the SRS light path to permit colocalization of the 2 modalities on the same microsphere. The resulting overlapping precision is within 500 nm. The prepared sample was first imaged with a 60× water immersion objective (UplanSAPO, NA 1.2). The brain tissue section was first imaged with fluorescence and then SRS. For both modalities, the multiarea time lapse function of the microscope was utilized to perform stitching that covers a large area of the brain tissue section.

Each section was imaged for fluorescence that labeled microglia and SRS that detects lipid. The images were reconstructed to create a large image area by home-built MATLAB code. To quantify the lipids associated with microglia, 2 masks were generated. In the SRS image, a manually selected area that covers the cortex was used to generate the mask. In the fluorescence image, a median filter was applied to the image, with a neighborhood size of 8 × 8 pixels. The filtered image was then binarized with Otsu’s method to generate a mask of each individual microglia. Finally, the 2 masks were combined to generate a mask of microglia located in the cortex of interest only. Then, the SRS and fluorescence images were divided into 50 × 50 pixel (about 25 μm × 25 μm) blocks. The mean lipid level of SRS blocks whose corresponding fluorescence blocks contain microglia (including part of a microglia) was then quantified.

### Behavioral assessment

The novel object recognition protocol included 3 phases: habituation, training, and object recognition. The habituation phase included 1 session, 5 minutes in length, in which the animals were allowed to freely explore a small Plexiglas arena (measuring 22 × 44 cm) that was utilized in the training and testing phase. The animals underwent training 1 day after habituation. During the training phase, the animals were placed in the same arena with the addition of 2 identical objects. The animals were allowed to freely explore the objects for 5 minutes. Twenty-four hours after the training phase, the test phase was initiated. During the testing phase, the animal was placed in the same arena with 1 object previously explored in the training phase, the familiar object, and 1 novel object differing in color and shape, but having a common size and volume. The animals were allowed to freely explore the objects for 5 minutes. ANY-Maze software (https://www.any-maze.com/) was used to measure time spent exploring each object. Exploration of an object was defined by head orientation directed toward the object or physical contact with the object.

### Statistics

All statistical analysis was performed using GraphPad Prism software v8.0.2. All data are presented as mean ± SEM. Unless otherwise noted, all group comparisons were made using 1-way ANOVA with Tukey’s correction, and all pairwise comparisons by 2-sided Student’s *t* tests, depending on experimental design. For all tests, *P* values less than 0.05 were considered significant, and those over 0.05 were considered nonsignificant: *P < 0.05, **P < 0.01, ***P < 0.001.

### Study approval

All animal procedures were performed in accordance with the NIH *Guide for the Care and Use of Laboratory Animal*s (National Academies Press, 2011) and with the approval of the Baylor College of Medicine Institutional Animal Care and Use Committee.

## Author contributions

MG and HZ conceived of the project and designed the experiments. MG performed all experiments and data analysis unless otherwise noted. MMC performed behavioral analysis. NEP assisted with the initial mouse generation and FACS analysis. TC performed in vivo SRS imaging and analysis, while FJ performed lipidomics and assisted the analysis, under guidance from MCW. MG wrote the manuscript with input and revision from HZ. All authors read and approved the final manuscript.

## Supplementary Material

Supplemental data

Supplemental tables 1-6

## Version 1 06/15/2023

Electronic publication
