# Supplementary material for: Complement C3aR depletion reverses HIF-1α–induced metabolic impairment and enhances microglial response to Aβ pathology
Source: J Clin Invest. 2023 Jun 15;133(12):e167501. doi: 10.1172/JCI167501 (PMC10266793; doi:10.1172/JCI167501)
Supplement: Supplemental data [file jci-133-167501-s234.pdf]

**Supplemental Materials**

**Supplemental Methods**

***Behavioral Assessments***

***Open field:*** The testing room is set up with white noise (~60 dB) and yellow lights prior to moving mice into the room. Mice are acclimated to the testing room for at least 30 minutes prior to testing. Mice are placed in Versamax animal activity chamber and allowed to freely explore for 30 minutes. Software (Veramax) collects total activity every 2 minutes for 15 time points to assess total distance travelled, movement time etc. Like an open field, for novel object recognition, the testing room is set up with white noise (~60 dB) and yellow lights prior to moving mice into the room. Mice are acclimated to the testing room for at least 30 minutes prior to testing.

***Fear conditioning:*** protocol was performed in three phases - The training phase, the context test and the cued test as previously described. Briefly, during the training phase the mice were placed in the training chamber and allowed to freely explore the environment. An 80-dB white noise was presented as auditory conditioned stimulus for 30 seconds at 3 minutes. During the last 2 seconds of the auditory stimulus, the unconditioned stimulus (US), a foot shock (0.8 mA, 2 seconds), was administered. The conditioned stimulus and unconditioned stimulus were then presented a second time at the 5-minute time point of the training procedure. After the second presentation of the unconditioned stimulus, the mice stayed in the training chamber for an additional 2 minutes without additional stimulation. The animals were returned to their original housing cages. 24 hours after the training procedure, the context test was performed. The mice were returned to the same training chamber consisting of the same context as the first procedure (same geometric shape of chamber, lights, scents and auditory sounds) for 3 minutes with no presentations of unconditioned stimulus or conditioned stimulus. One hour later, the cue test was performed. The cue test chamber consisted of a different geometric shape, flooring, light brightness and scent compared to the previous chamber used for training. After 3 minutes in the chamber, the auditory stimulus was presented for 3 minutes. The software, FreezeFrame3 and FreezeView (San Diego Instruments) was used to record and analyze the percent freezing in each trial.

## Supplemental Figures and Figure Legends

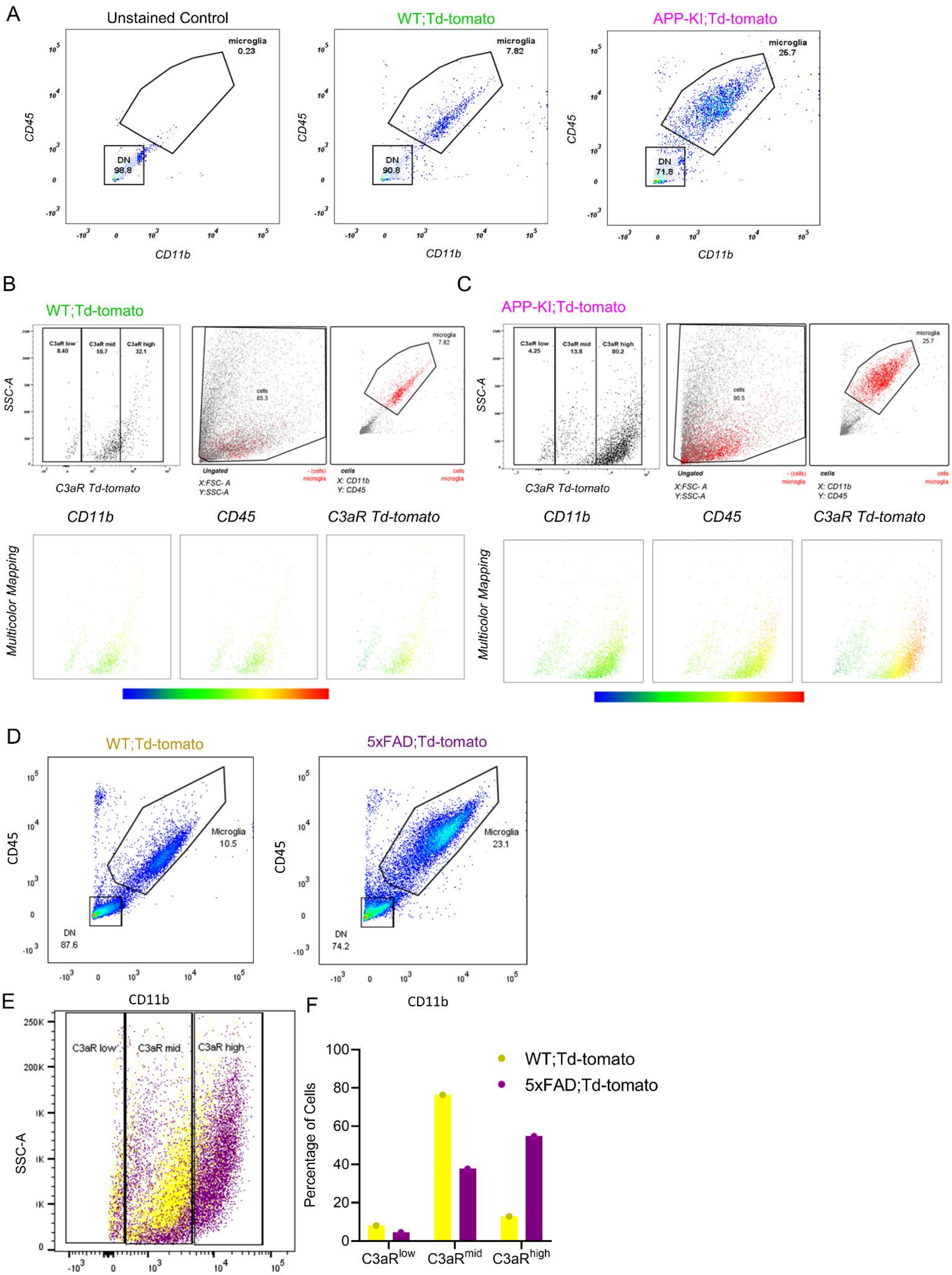
A
Unstained Control
WT;Td-tomato
APP-KI;Td-tomato
microglia 0.23
microglia 7.82
microglia 25.7
DN 98.8
DN 90.8
DN 71.8
CD45
CD11b
B
C
WT;Td-tomato
APP-KI;Td-tomato
C3aR low 8.40
C3aR mid 59.7
C3aR high 32.1
C3aR low 4.25
C3aR mid 13.8
C3aR high 80.2
SSC-A
C3aR Td-tomato
cells 85.3
cells 90.5
Ungated
X:FSC- A
Y:SSC-A
- (cells) microglia
cells
X: CD11b
Y: CD45
cells microglia
CD11b
CD45
C3aR Td-tomato
Multicolor Mapping
D
WT;Td-tomato
5xFAD;Td-tomato
Microglia 10.5
Microglia 23.1
DN 87.6
DN 74.2
CD45
CD11b
E
C3aR low
C3aR mid
C3aR high
SSC-A
CD11b
F
WT;Td-tomato
5xFAD;Td-tomato
Percentage of Cells
C3aR^low
C3aR^mid
C3aR^high

**Supplemental Figure 1. Characterization of C3aR expression in APP-KI and 5xFAD mice.**

**(A)** Flow cytometry analysis showing the gating strategy to identify microglial population in 9-month-old samples from unstained control, WT;Td-tomato and APP-KI;Td-tomato mice. **(B)** Ancestry and backgated flow plots for 9-month-old WT;Td-tomato animal showing the final gated population in C3aR Td-tomato gates within the population of its ancestors (in red). Multicolor mapping showing the intensity of signal from CD11b, CD45 and C3aR Td-tomato expression. The color scaling axis from blue (low) to red (high). **(C)** Ancestry and backgated flow plots for 9-month-old APP-KI;Td-tomato animal showing the final gated population in C3aR Td-tomato gates within the population of its ancestors (in red). Multicolor mapping showing the intensity of signal from CD11b, CD45 and C3aR Td-tomato expression. The color scaling axis from blue (low) to red (high). **(D)** Flow cytometry plots from 11 month-old 5xFAD;Td-tomato mice and its littermate WT;Td-tomato mice. **(E)** Flow cytometry plot showing the distribution of microglia from WT (yellow) and 5xFAD (purple) mice based on C3aR expression. **(F)** Bar graph showing the percentage of C3aR expression in WT and 5xFAD mice.

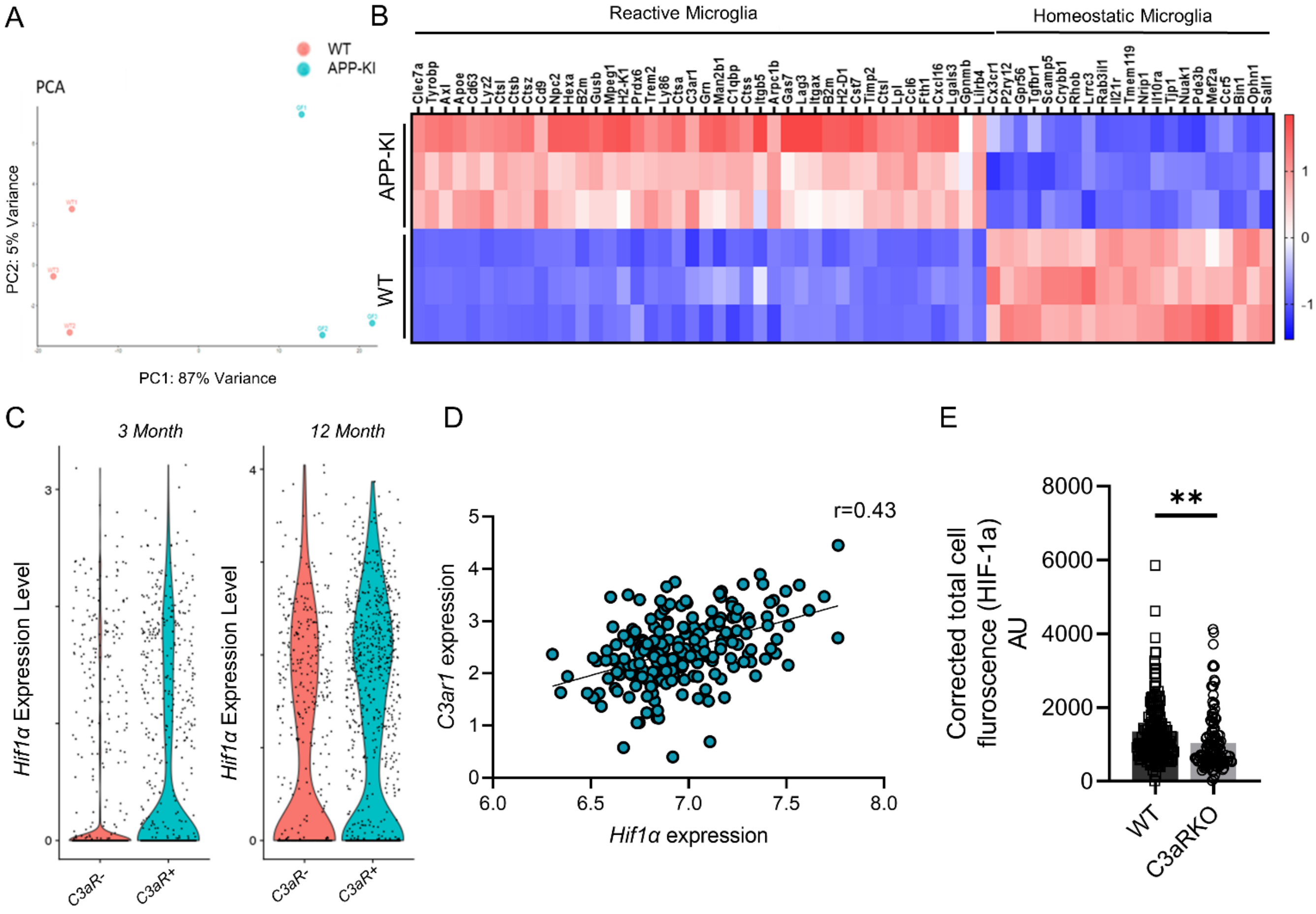


**Supplemental Figure 2. C3aR and HIF-1α expression in microglia of WT and APP-KI mice.**

**(A)** Principal component analysis plot showing the separation C3aR positive cells from WT and APP-KI mice. **(B)** Heatmap depicting the genes associated with microglial homeostatic and reactive states and the altered expression profile in APP-KI mice compared to WT. **(C)** Volcano plots from single cell sequencing data from showing the distribution of HIF-1a expression in $C3aR^+$ and $C3aR^-$ microglia population in 3- and 12-months-old APP-KI mice**. (D)** Correlation between *C3ar1* and *HIF-1α* expression in human AD patients from Mount Sinai School of Medicine, AMP-AD RNA-seq project (Synapse ID: syn8484987). **(E)** Quantification showing corrected total cell fluorescence (CTCF) of HIF-1α from primary microglial cells represented in Figure 2A, where each dot represents an individual cell. Data presented as mean ± SEM. ***P* < 0.01 by 2-sided *t*-tests.

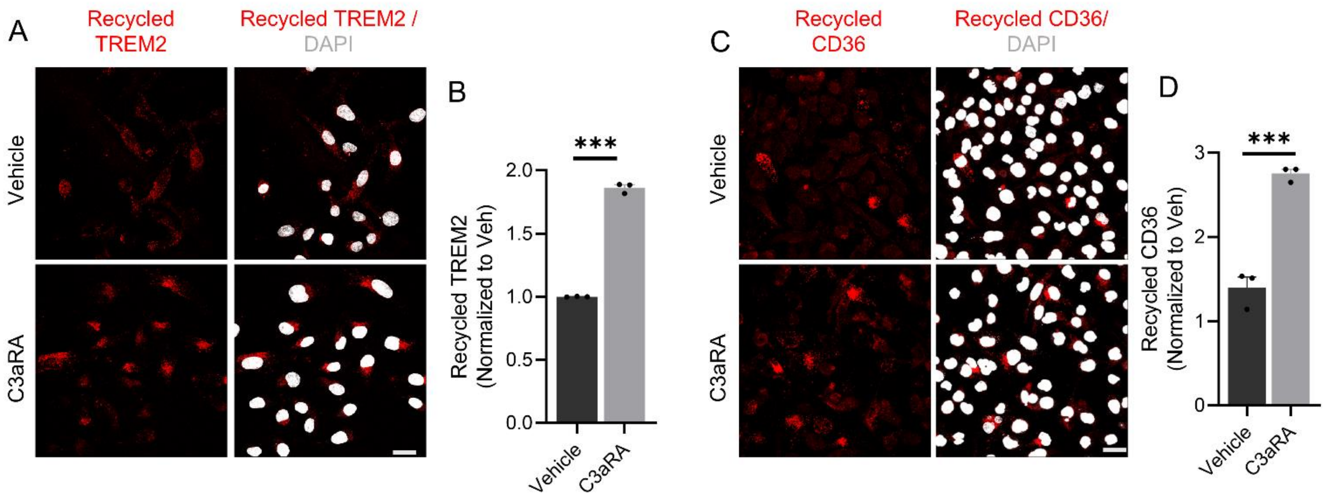


**Supplemental Figure 3. C3aR antagonist treated BV-2 cells have improved phagocytic receptor recycling.**

**(A)** Representative images of TREM2 recycling in Vehicle and 10 μM C3aRA treated BV-2 cells. Scale Bar: 15 μm. **(B)** Quantification of TREM2 recycling. **(C)** Representative images of CD36 recycling in Vehicle and 10 μM C3aRA treated BV-2 cells. Scale bar: 15 μm. **(D)** Quantification of (C). For all panels, data are presented as mean ± SEM. ***$P$ < 0.001 by 2-sided $t$-tests.

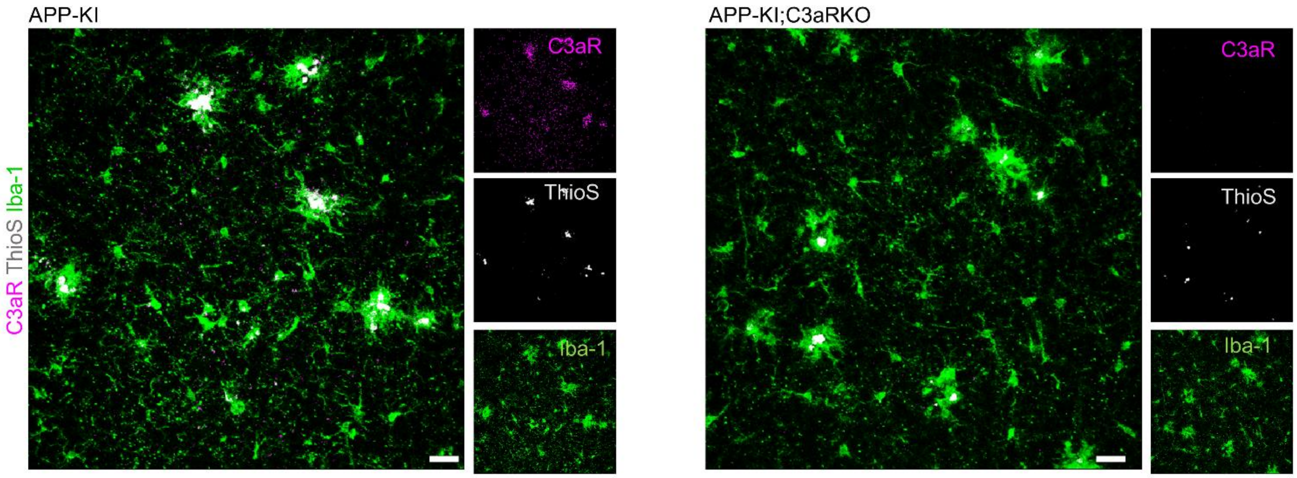


**Supplemental Figure 4. Validation of C3aR ablation in APP-KI mice.**

Representative images from 9-month-old APP-KI and APP-KI; C3aRKO sections stained for C3aR (magenta), Thioflavin S (grey) and Iba-1 (green). Scale bar: 50 μm.

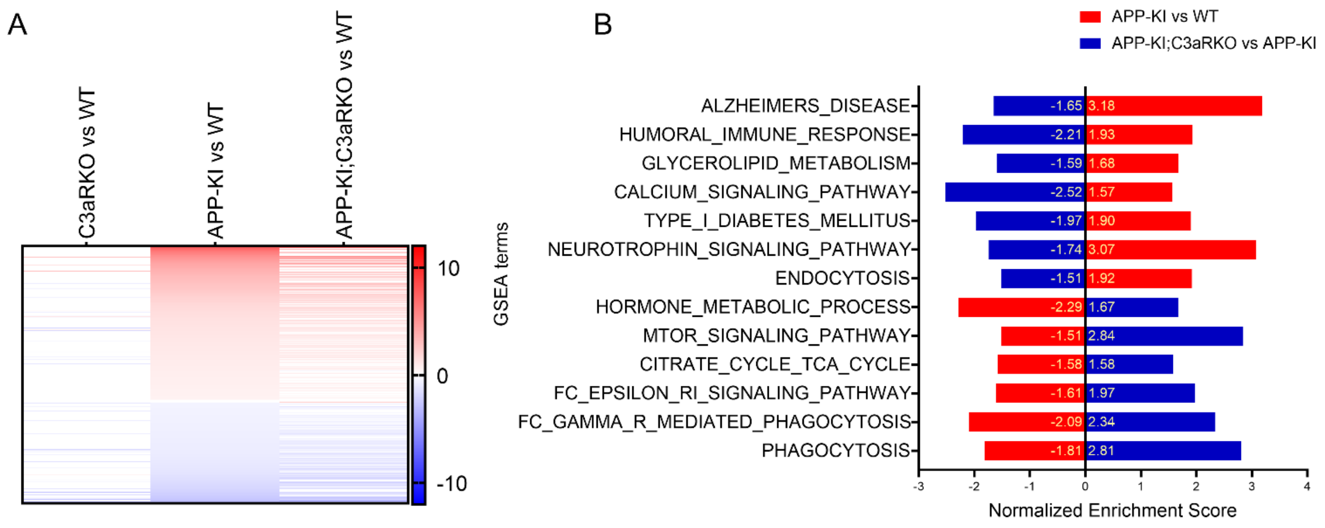


**Supplemental Figure 5. Altered metabolic profile in APP-KI; C3aRKO compared to APP-KI.**

**(A)** Heatmap showing two-way comparisons of significant DEGS identified from 9-month-old sorted microglia from WT, C3aRKO, APP-KI and APP-KI; C3aRKO animals. **(B)** Gene set enrichment analysis results depicted with APP-KI over WT comparisons (red) to APP-KI; C3aRKO over APP-KI comparisons (blue). Gene set enrichment terms from both KEGG as well as GO analysis. Number within the bars indicate normalized gene set enrichment score of the term.

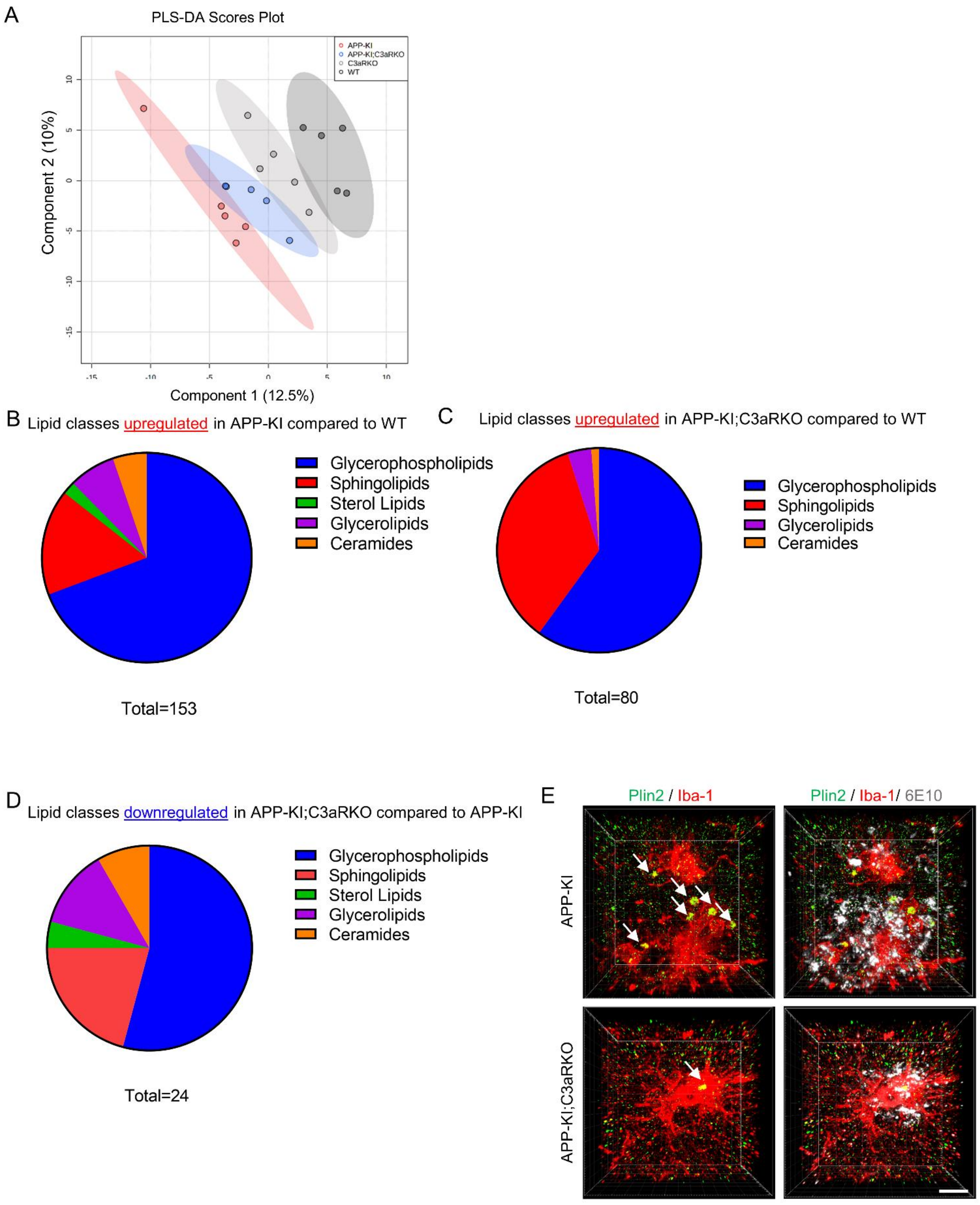
A
PLS-DA Scores Plot
APP-KI
APP-KI;C3aRKO
C3aRKO
WT
Component 2 (10%)
Component 1 (12.5%)
B Lipid classes upregulated in APP-KI compared to WT
Glycerophospholipids
Sphingolipids
Sterol Lipids
Glycerolipids
Ceramides
Total=153
C Lipid classes upregulated in APP-KI;C3aRKO compared to WT
Glycerophospholipids
Sphingolipids
Glycerolipids
Ceramides
Total=80
D Lipid classes downregulated in APP-KI;C3aRKO compared to APP-KI
Glycerophospholipids
Sphingolipids
Sterol Lipids
Glycerolipids
Ceramides
Total=24
E
Plin2 / Iba-1
Plin2 / Iba-1/ 6E10
APP-KI
APP-KI;C3aRKO

**Supplemental Figure 6. Deletion of *C3ar1* dampens lipid dysregulation in APP-KI mice.**

**(A)** PLS-DA plot showing the separation of lipid profiles of cortical tissues of 9-month-old WT, C3aRKO, APP-KI and APP-KI; C3aRKO mice. **(B)** Pie chart showing the lipid classes upregulated in APP-KI mice compared to WT (Total = 153 lipids). **(C)** Pie chart showing lipid classes upregulated in APP-KI; C3aRKO compared to WT (Total= 80 lipids). (D) Pie chart showing lipid classes downregulated in APP-KI; C3aRKO compared to APP-KI (Total= 24 lipids). (E**)** Representative immunostaining of Plin2 (green), Iba-1 (red) and 6E10 (grey) from 9-month old APP-KI and APP-KI;C3aRKO mice. Scale bar: 10 $\mu$m.

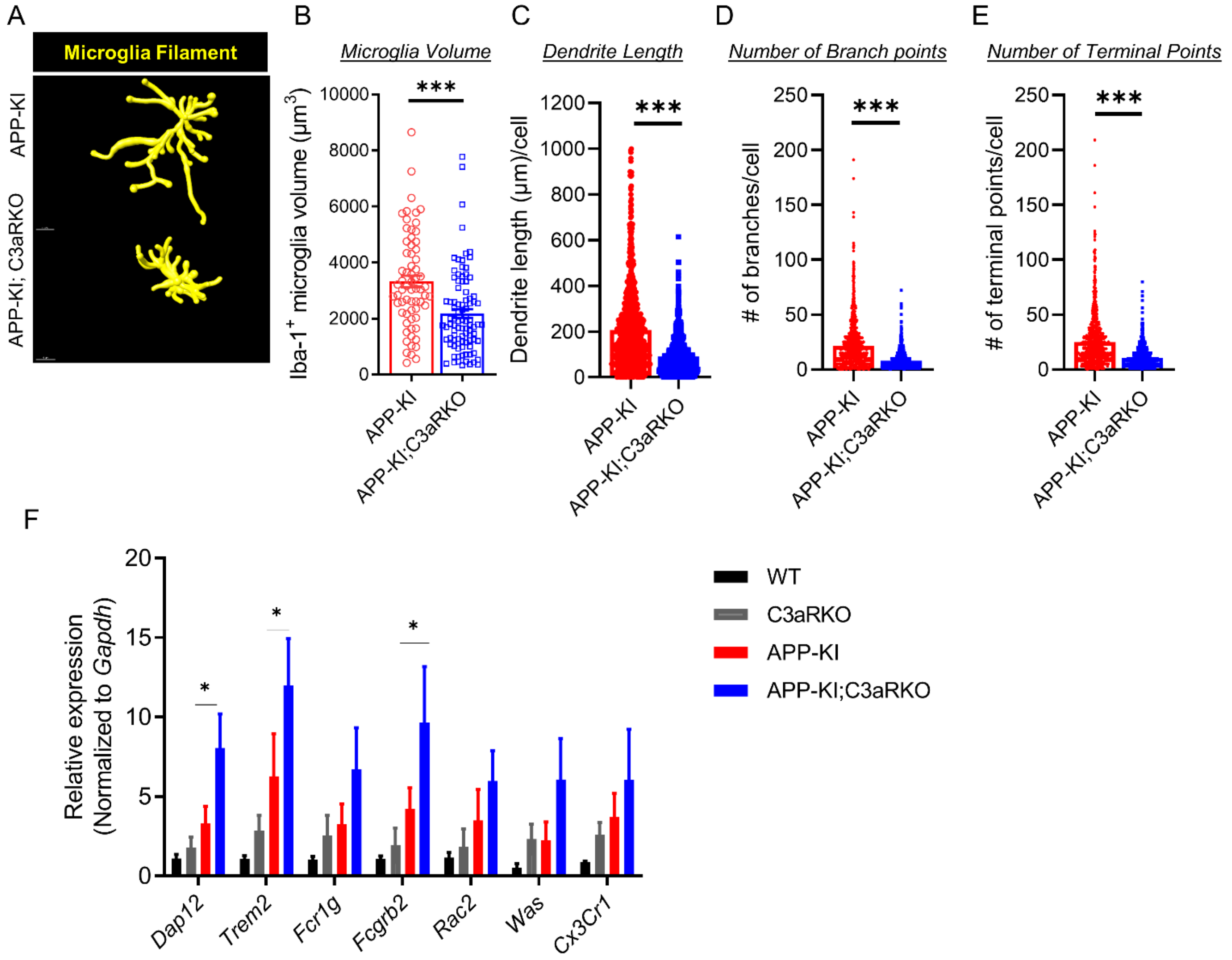


**Supplemental Figure 7. Characterization of microglial morphology and phagocytic markers.**

**(A)** Representative images of microglia showing 3D skeletons of microglial filaments. Scale bar: 4 μm. Quantification of microglia in APP-KI and APP-KI; C3aRKO showing differences in **(B)** microglia volume and microglia morphology in terms of **(C)** dendrite length, **(D)** number of branch points and **(E)** number of terminal points. **(F)** Bar graph showing relative expression of genes involved in phagocytosis from cortical lysates of 9-month-old animals from WT, C3aRKO, APP-KI and APP-KI; C3aRKO. For all panels, data are presented as mean ± SEM. $^*P < 0.05$, $^{***}P < 0.001$ by 2-sided *t*-tests (B-E) or one-way ANOVA (F).

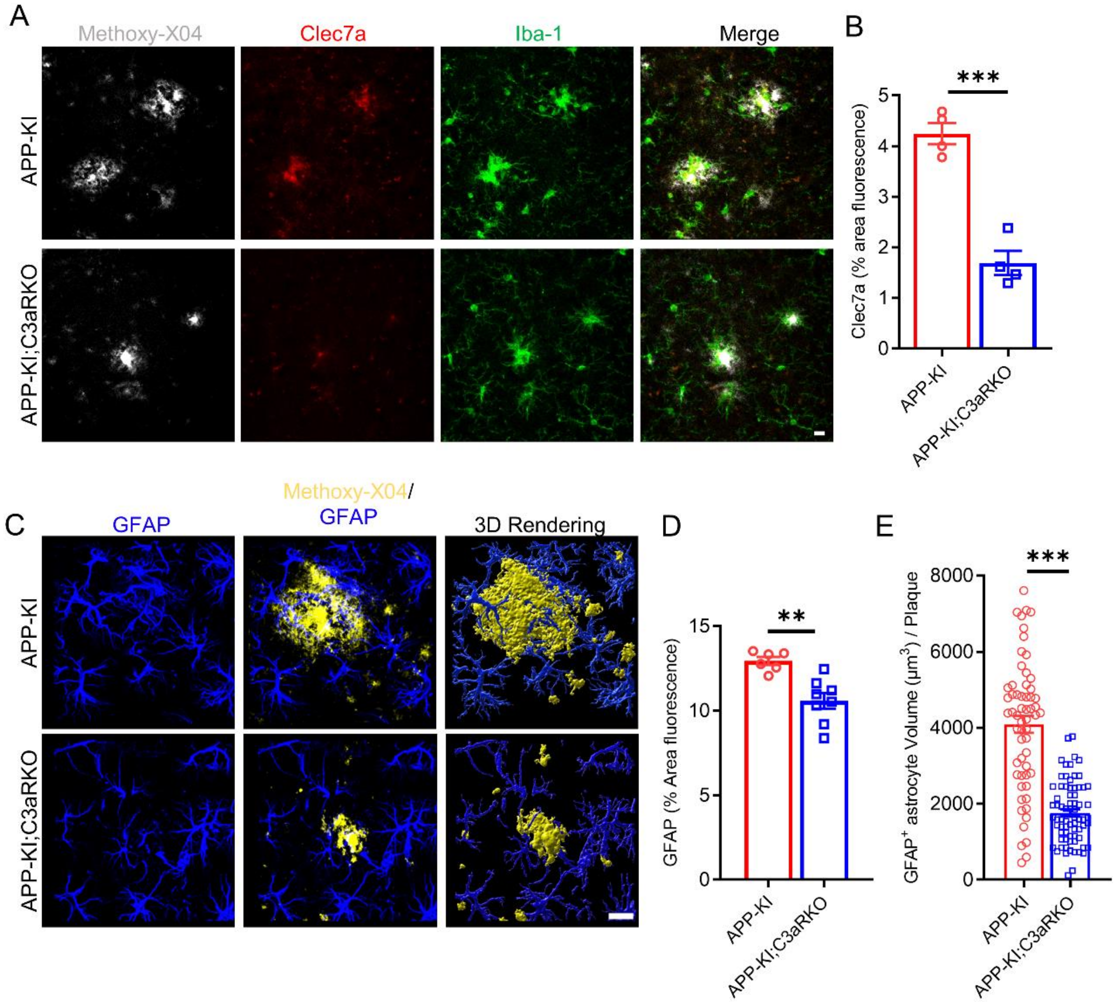


**Supplemental Figure 8. Deletion of *C3ar1* reduces neuroinflammation in microglia and astrocytes.**

**(A)** Representative images from 9-month-old APP-KI and APP-KI; C3aRKO showing Methoxy XO-4 (grey), Clec7a (red), Iba-1 (green) expression. Scale bar: 15 μm. **(B)** Quantification of Clec7a expression in (A). **(C)** Representative images of Methoxy XO-4 (yellow) and GFAP (blue) and 3D surface reconstruction from APP-KI and APP-KI; C3aRKO cortical tissue. Scale bar: 15 μm (*n*=4 animals/group) **(D)** Quantification of GFAP expression in 9-month-old animals. (*n*=7-8 animals/group). **(E)** Quantification of $GFAP^+$ astrocyte volume surrounding plaques in panel C. For all panels, data are presented as mean ± SEM. $^{**}P < 0.01$, $^{***}P < 0.001$ by 2-sided *t*-tests.

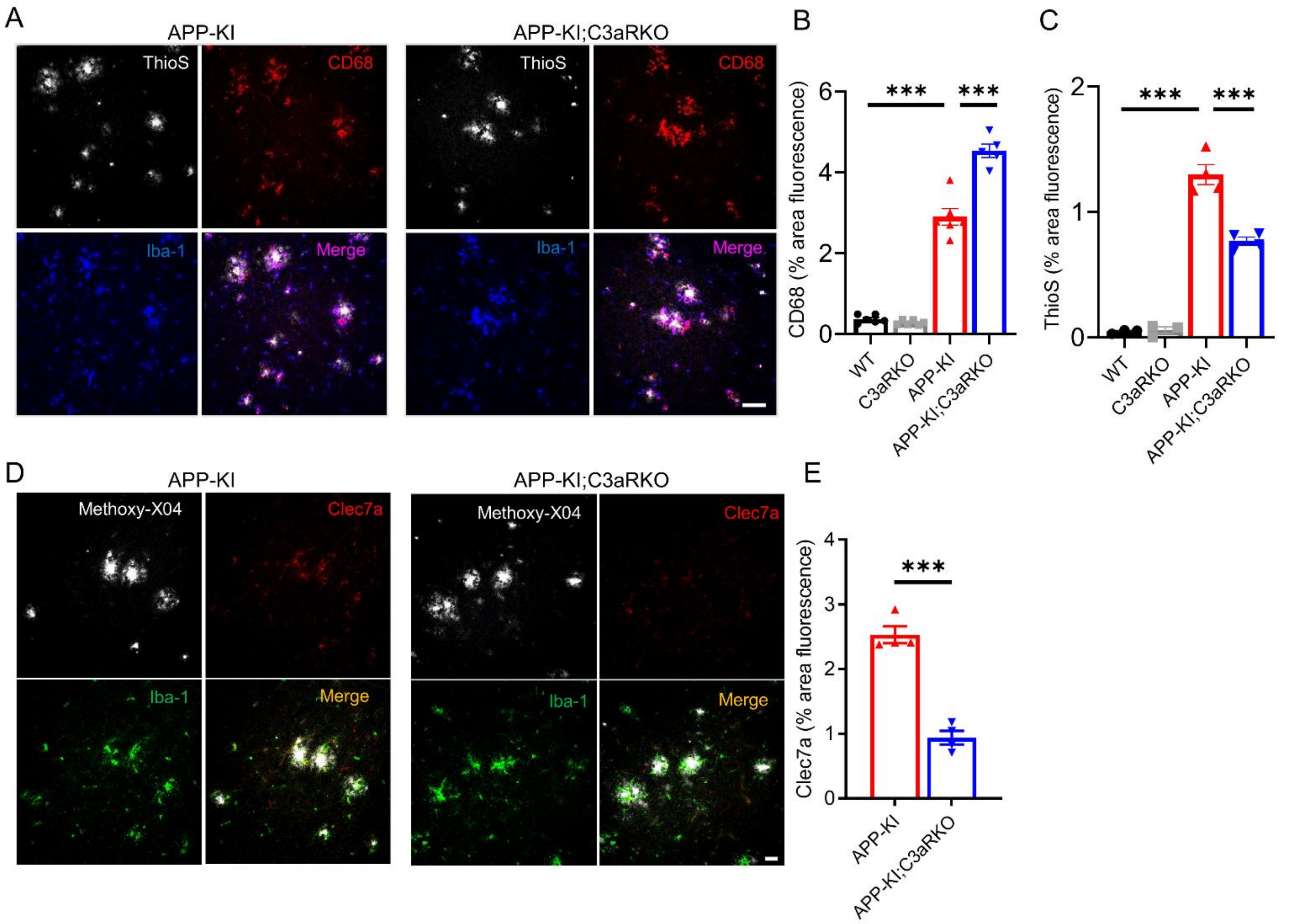


**Supplemental Figure 9. Deletion of *C3ar1* improves microglial phagocytosis and reduces Clec7a and Aβ pathology in 6-month-old mice.**

**(A)** Representative images showing the expression of Thioflavin S (grey), CD68 (red) and Iba-1 (blue) in 6-month-old animals from APP-KI and APP-KI; C3aRKO. Scale bar: 50 μm. **(B)** Quantification of percent area fluorescence of CD68. **(C)** Quantification of percent area fluorescence of ThioS *(n*=5-6 mice/group*).* **(D)** Representative images of Methoxy- XO4 (grey), Clec7a (red), Iba-1 (green) in APP-KI and APP-KI; C3aRKO brain sections. **(E)** Quantification of percent area fluorescence of Clec7a from panel (D). (*n*=4 mice/group). Data presented as mean ± SEM. ****P* < 0.001 by 2-sided *t*-tests (E) or one-way ANOVA (B and C).

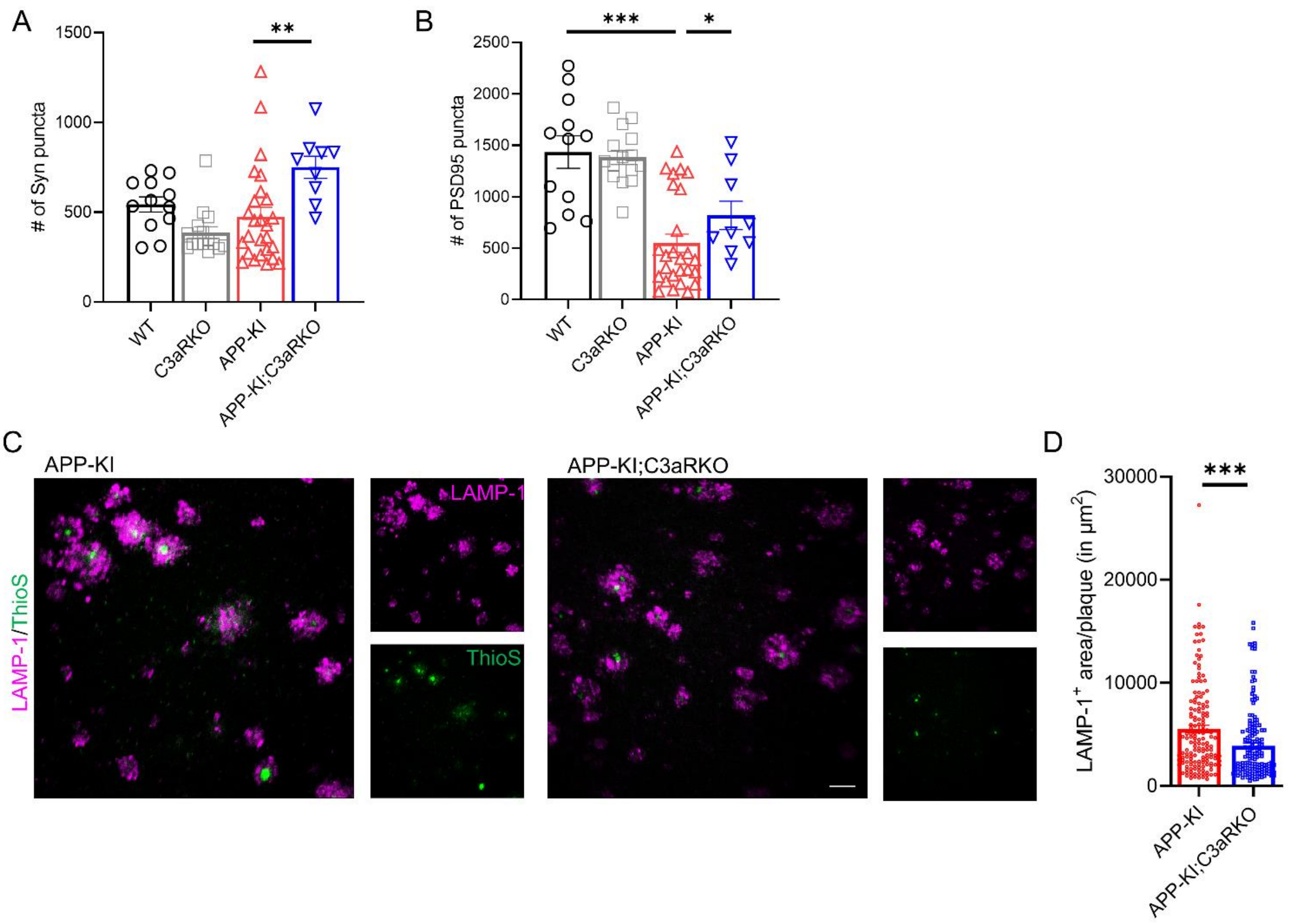


**Supplemental Figure 10. Deletion of *C3ar1* rescues dystrophic neurites in 9-month-old mice.**

**(A)** Total number of synaptophysin puncta in 9-month animals across 4 genotypes. **(B)** Total number of PSD95 puncta in 9-month-old animals from 4 genotypes. **(C)** Representative images from tissues stained for Lamp-1 to mark dystrophic neurites in 9-month-old APP-KI and APP-KI; C3aRKO animals. Scale bar: 100 µm. **(D)** Quantification of Lamp1+area/plaque in APP-KI and APP-KI; C3aRKO animals. For all panels, data are presented as mean ± SEM. *$P < 0.05$, **$P < 0.01$, ***$P < 0.001$ by 2-sided *t*-tests (D) or one-way ANOVA (A and B).

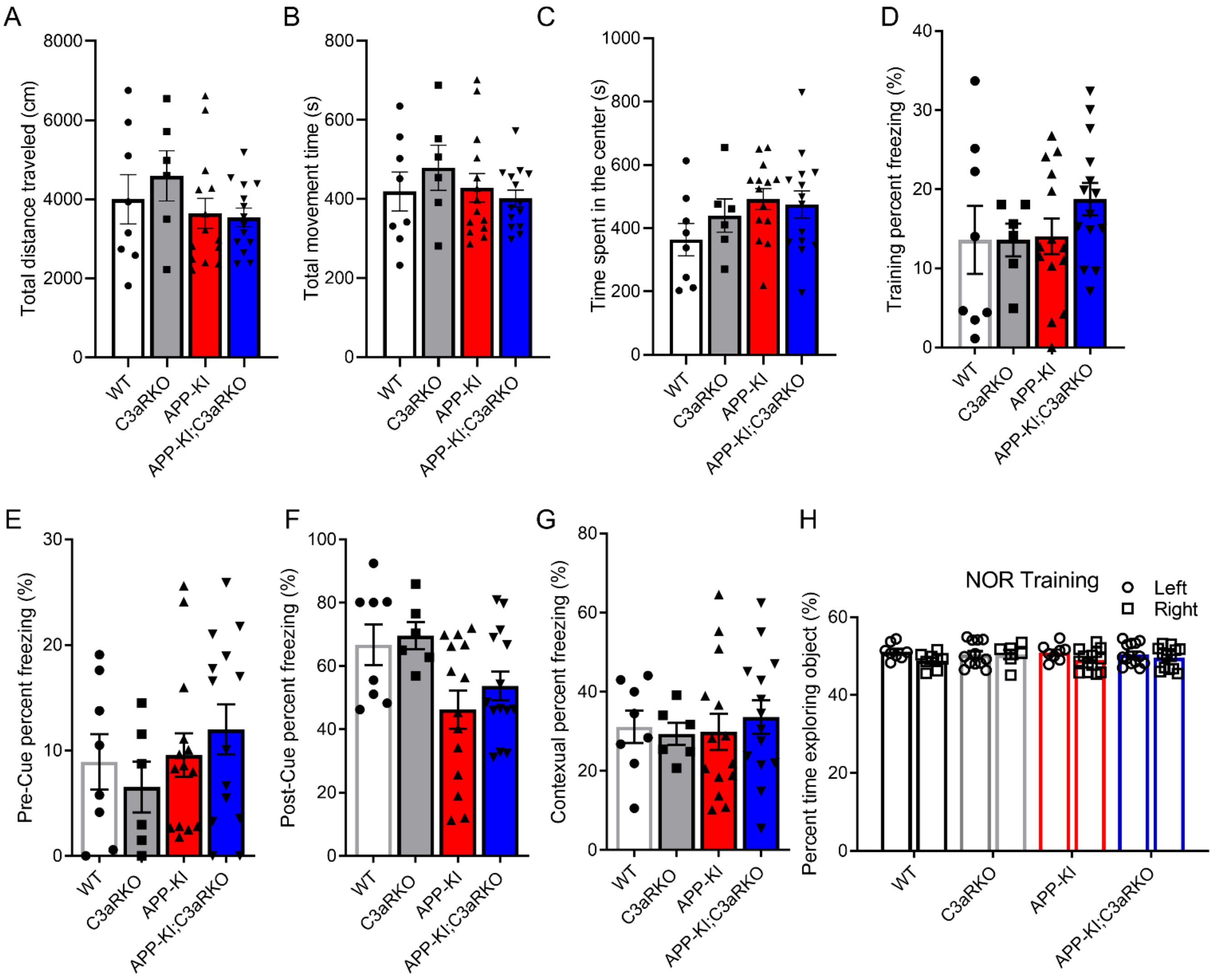


**Supplemental Figure 11. Deletion of *C3ar1* does not affect mobility or anxiety in 9-month-old mice. (A-C)** Bar graphs showing no significant differences in total distance travelled **(A)**, total movement time **(B)**, and time spent in the center **(C)** during open field behavioral test. **(D-G)** Bar graphs showing no significant differences in training **(D)**, pre-cue **(E),** post-cue **(F)** or contextual testing **(G)** during fear-conditioning behavioral test. **(H)** Bar graph showing no significant differences in training phase of novel object recognition. One-way ANOVA with Tukey's correction. For all panels, data are presented as mean ± SEM.

**Summary of Supplemental Tables (contents of each table is included in a separate Excel file)**

| | |
|---|---|
| **Supplemental Table 1** | DE-Seq2 results from C3aR positive microglial cells sorted from 9-month-old WT and APP-KI mice |
| **Supplemental Table 2** | Gene list and Z-score of genes included in heatmap for hypoxia mediated module (HMM), lipid metabolism and microglial reactivity |
| **Supplemental Table 3** | Primer list |
| **Supplemental Table 4** | List of differential lipids in APP-KI compared to WT |
| **Supplemental Table 5** | List of altered lipid species in APP-KI compared to WT and Z-score across 4 genotypes |
| **Supplemental Table 6** | List of antibodies and dyes |
